# Effects of Regular Physical Activity on the Immune System, Vaccination and Risk of Community-Acquired Infectious Disease in the General Population: Systematic Review and Meta-Analysis

**DOI:** 10.1007/s40279-021-01466-1

**Published:** 2021-04-20

**Authors:** Sebastien F. M. Chastin, Ukachukwu Abaraogu, Jan G. Bourgois, Philippa M. Dall, Jennifer Darnborough, Elaine Duncan, Jasmien Dumortier, David Jiménez Pavón, Joanna McParland, Nicola J. Roberts, Mark Hamer

**Affiliations:** 1 School of Health and Life Science, Glasgow Caledonian University, Cowcaddens Rd, Glasgow, G4 0BA UK; 2 Department of Movement and Sports Sciences, Ghent University, Ghent, Belgium; 3 Department of Public Health, NHS Lanarkshire, North Lanarkshire, Scotland, UK; 4 MOVE-IT Research Group, Department of Physical Education, Faculty of Education Sciences, University of Cádiz, Cádiz, Spain; 5 Biomedical Research and Innovation Institute of Cádiz (INiBICA) Research Unit, Puerta del Mar University Hospital University of Cádiz, Cádiz, Spain; 6 Institute Sport Exercise and Health, Division of Surgery and Interventional Science, Faculty Medical Sciences, University College London, London, UK

## Abstract

**Background:**

Regular physical activity is the prime modality for the prevention of numerous non-communicable diseases and has also been advocated for resilience against COVID-19 and other infectious diseases. However, there is currently no systematic and quantitative evidence synthesis of the association between physical activity and the strength of the immune system.

**Objective:**

To examine the association between habitual physical activity and (1) the risk of community-acquired infectious disease, (2) laboratory‐assessed immune parameters, and (3) immune response to vaccination.

**Methods:**

We conducted a systemic review and meta-analysis according to PRISMA guidelines. We searched seven databases (MEDLINE, Embase, Cochrane CENTRAL, Web of Science, CINAHL, PsycINFO, and SportDiscus) up to April 2020 for randomised controlled trials and prospective observational studies were included if they compared groups of adults with different levels of physical activity and reported immune system cell count, the concentration of antibody, risk of clinically diagnosed infections, risk of hospitalisation and mortality due to infectious disease. Studies involving elite athletes were excluded. The quality of the selected studies was critically examined following the Cochrane guidelines using ROB2 and ROBINS_E. Data were pooled using an inverse variance random-effects model.

**Results:**

Higher level of habitual physical activity is associated with a 31% risk reduction (hazard ratio 0.69, 95% CI 0.61–0.78, 6 studies, *N* = 557,487 individuals) of community-acquired infectious disease and 37% risk reduction (hazard ratio 0.64, 95% CI 0.59–0.70, 4 studies, *N* = 422,813 individuals) of infectious disease mortality. Physical activity interventions resulted in increased CD4 cell counts (32 cells/µL, 95% CI 7–56 cells/µL, 24 studies, *N* = 1112 individuals) and salivary immunoglobulin IgA concentration (standardised mean difference 0.756, 95% CI 0.146–1.365, 7 studies, *N* = 435 individuals) and decreased neutrophil counts (704 cells/µL, 95% CI 68–1340, 6 studies, *N* = 704 individuals) compared to controls. Antibody concentration after vaccination is higher with an adjunct physical activity programme (standardised mean difference 0.142, 95% CI 0.021–0.262, 6 studies, *N* = 497 individuals).

**Conclusion:**

Regular, moderate to vigorous physical activity is associated with reduced risk of community-acquired infectious diseases and infectious disease mortality, enhances the first line of defence of the immune system, and increases the potency of vaccination.

**Protocol registration:**

The original protocol was prospectively registered with PROSPERO (CRD42020178825).

**Supplementary Information:**

The online version contains supplementary material available at 10.1007/s40279-021-01466-1.

## Key Points

**Table:** 

Engaging regularly in moderate to vigorous physical activity is associated with a 31% risk reduction of community-acquired infectious disease and 37% risk reduction in infectious disease mortality
Engaging regularly in moderate to vigorous physical activity is associated with the increased strength of the mucosal immune barrier (salivary IgA immunoglobulin) and higher concentration of immune cells that prepare, orchestrate, regulate and effect immunity (CD4 T-cells)
Engaging regularly in moderate to vigorous physical activity could strengthen the effect of vaccination campaigns

## Introduction

Lower respiratory tract infections and pneumonia account for more than 4 million deaths annually and upper respiratory infections rank as the leading incident disease in the world [1]. These infections are caused by viruses or a combination of viral and bacterial invasion. They can be very contagious and spread rapidly leading to epidemics and pandemics as in the case of SARS-CoV-2 (COVID-19). There are numerous public health strategies to cope with pandemics [2]. An important approach to containing the recent SARS-CoV-2 (COVID-19) outbreak has been the requirement of communities to remain at home (“lockdown”) thereby reducing social contact and containing the spread of the virus. Despite these restrictions, numerous governments underlined the importance of remaining physically active for health and wellbeing [3], and recognised the necessity to allow individuals to leave their home to walk, cycle or run.

Regular physical activity is associated with the prevention of numerous non-communicable diseases [4]. Physical activity may also have important functions in a pandemic and in the prevention of infectious diseases. Firstly, it has been hypothesised that physically active people are likely to be more resilient to infection through better immunosurveillance against pathogens [5]. Secondly, given that severe infections are more likely in individuals with poorer cardiovascular and metabolic health and who might have pre-existing chronic conditions, we might hypothesise that physical activity also has an indirect protective effect against infectious disease by improving cardiovascular and metabolic health and lowering the risk of chronic diseases [6, 7]. Third, there is a suggestion that physical activity is also associated with a more favourable response to vaccination [6]; thus acquired immunity could be greater in a physically active population.

Some have advocated the importance of physical activity for resilience against COVID-19 [6, 8–11]. Strong claims have been made in support of physical activity and its therapeutic effects on immunity [5] but as yet, there has been no attempt to systematically evaluate the current evidence on the effect of habitual physical activity on laboratory‐assessed immune parameters, and risk of community-acquired infectious disease for the general population based on objective markers. A number of narrative reviews have debated the effect of physical activity on human immunity [5, 12] but the majority of this literature is devoted to the acute effect of exercises and focuses on athletes. Some reviews and meta-analysis have evaluated the impact of exercise on the risk of self-reported upper respiratory tract infection, but these were inconclusive due to a low level of evidence and very few available studies [13, 14].

There is a need for clear evidence, based on objective markers, on the importance of engaging in regular physical activity for the general population that will help develop a policy for the ongoing COVID-19 outbreak. This will be crucial to help populations better fight this virus during a possible future wave, maximise responses to vaccination programmes when available, and inform preparation for future pandemics.

We aimed to perform a meta-analysis of randomised controlled trials (RCTs) and prospective studies to answer the following questions:

1. Does engaging in habitual physical activity decrease the risk of community-acquired infectious disease and related mortality?
2. Does engagement in physical activity improve immunosurveillance and response?
3. Does engagement in physical activity increase the effect of immunisation?

## Methods

We conducted this systematic review and meta-analysis according to the PRISMA guidelines [15]. We prospectively registered the review with PROSPERO (CRD42020178825) but made alterations (described below) to focus this meta-analysis on the chronic effect of physical activity.

### Search Strategy

We searched seven databases: MEDLINE, Embase, Cochrane CENTRAL, Web of Science, CINAHL, PsycINFO, and SportDiscus from January 1980 until 14 April 2020 for peer-reviewed journal articles published in the English language. The search strategy is presented in Electronic Supplementary Material (ESM) Table S1. In addition, we complemented the search by scanning the reference list of full texts included and relevant systematic reviews.

### Criteria for Considering Studies for This Review

#### Types of Studies

We included observational studies (prospective cohort studies) and RCTs including cluster RCTs in humans. We excluded controlled before-after (CBA) and non-randomised experimental studies, qualitative studies, reviews, opinion pieces and cross-sectional and case controlled observational studies. If studies had duplicated data, we included only one study. We selected this based on risk of bias, sample size and date of publication. We excluded animal studies.

#### Types of Participants/Population

We included studies with adult participants who were ≥ 18 years. We excluded studies that compared outcomes in athletes and highly trained sports men and women, as such individuals undertake volumes and intensities of training unrepresentative of the general population.

#### Types of Interventions/Exposure

We included studies examining levels of habitual physical activity or regular exercise programmes. Studies where participants received multiple interventions were only included if the only difference between the groups was the physical activity intervention. We excluded studies that only used physical activity as a confounding variable or that did not present data on physical activity separately. We also excluded studies assessing the effect of a single bout of exercise or exercise session.

#### Comparator

We included studies that had no physical activity or a lesser volume, duration, frequency, or intensity of physical activity as a comparator.

#### Types of Outcome Measures

We focussed on chronic response to physical activity and excluded studies which reported only acute response to exercise. We included studies that reported objective measurement of at least one of these outcomes: immune system cell count such as white blood cell count, concentration of antibodies considered markers of depressed immune system, risk of clinically diagnosed infections (recorded by clinician), risk of clinically recorded complications (hospitalisation, mortality). We specifically excluded studies exclusively concerned with cytokines (interleukin-6), C-reactive protein (CRP), tissue necrotic factors, and other markers of inflammatory responses which are not immune cells. There are already several meta-analyses on the relationship between physical activity and inflammatory markers [16, 17]. We also excluded studies which used a self-reported measure of infection, hospitalisation or sequelae such as patients’ self-reported symptoms of upper respiratory tract infections.

### Screening

We imported the studies identified from the search into COVIDENCE (Veritas Health Innovation) to remove duplicates and for transparent management of the review by the team. Two review authors (from a pool of 11) independently screened studies and judged their eligibility for inclusion in the review. We resolved disagreements by discussion and consultation with a third review author when needed. We sought additional information from authors of studies when the information was inadequate to determine eligibility in the review.

### Data Extraction

Two review authors (from the same pool of 11) independently extracted data and resolved disagreements on data extraction including the results in the review, by discussion among the review team. When details were missing, we contacted the authors of the study to obtain the information.

### Assessment of Risk of Bias and Quality of Evidence Rating

Two authors from the same pool of 11 independently assessed the risk of bias in included studies with disagreement resolved by discussion or additional consultation with a third review author. We used ROB2 [18] to assess the risk of bias in RCTs and ROBINS-E [19] for observational studies.

### Quantitative Synthesis

We conducted separate meta-analyses for each outcome of RCTs and observational studies when studies provided outcome data in comparable units or effect size that could be pooled. We stratified the analysis by population groups when sufficient studies were available. These groups included healthy adults, obese individuals, older adults (aged 60 years and over) and clinical populations particularly at risk of infectious diseases such as HIV, organ transplant, and cancer patients. We computed the pooled effect size using random effect models as we hypothesised that a range of true effects was likely. For RCTs, we used the post-trial values for exercise and control groups rather than pre-post effect size to minimise bias [20]. When studies reported more than one-time point, we used data recorded immediately post-intervention. If trials included more than one intervention group, we included each intervention in a separate comparison. We divided the number of participants in the control group accordingly to avoid double counting [21]. When measurement methods of the outcome were similar, we computed the unstandardised mean difference (MD) in immune cell concentration. Otherwise, we calculated the standardised mean difference (SMD). However, if only a small minority of studies reported in different units, we favoured reporting the mean difference and excluded these studies. Heterogeneity was measured using *I*^2^ statistics. We assessed publication bias by inspecting funnel plots visually. When sufficient studies were available, we conducted meta-regression to estimate the moderating effect of frequency, intensity, type (aerobic or resistance training) and duration of physical programmes and to identify sources of heterogeneity. Finally, we conducted a sensitivity analysis to ascertain the robustness of the results. All meta-analyses were performed with random effect models using the Comprehensive Meta-Analysis Software (Version 3.3.07, Biostat, Englewood NJ).

### Deviation from Registered Protocol

This systematic review and meta-analysis includes some deviations and changes compared to the initial protocol registered on PROSPERO. The initial protocol was very ambitious and designed to synthesise all aspects of current research on the relationship between physical activity and human immunity and provide detailed quantitative synthesis to inform public health measures. The study was narrowed to focus on research on the chronic effect of regular physical activity as this is more directly relevant to population health using the highest level of evidence. Consequently, the following changes were made:

- Review questions: aim to synthesise the acute effect of physical activity was abandoned to focus the review on chronic effects of regular physical activity.
- Eligible studies: design was restricted to RCTs for experimental studies and prospective studies for observational studies as they constitute the highest level of evidence.
- Population: studies on elite athlete and highly trained individuals were excluded to focus on information more directly relevant to the general population who mostly engage in a low level of physical activity.
- Interventions: studies on single bouts of exercise were excluded to focus the review on information about regular physical activity.
- Outcomes: the list of outcomes was narrowed to objective markers of the immune system (cell counts) and objective records of infection and death due to infectious disease. Thus, for example, self-reported cases of upper respiratory tract infection were excluded.
- Risk of bias: we used risk of bias tools more widely recognised and more specific to the design included rather than a generic tool (Qualsys).
- Sub-group analysis: additional sub-group analyses were conducted (obesity, clinical populations) to analyse heterogeneity and understand the potential modifying effect of well known factors for which data existed.

## Results

### Study Characteristics

**Fig. 1:**
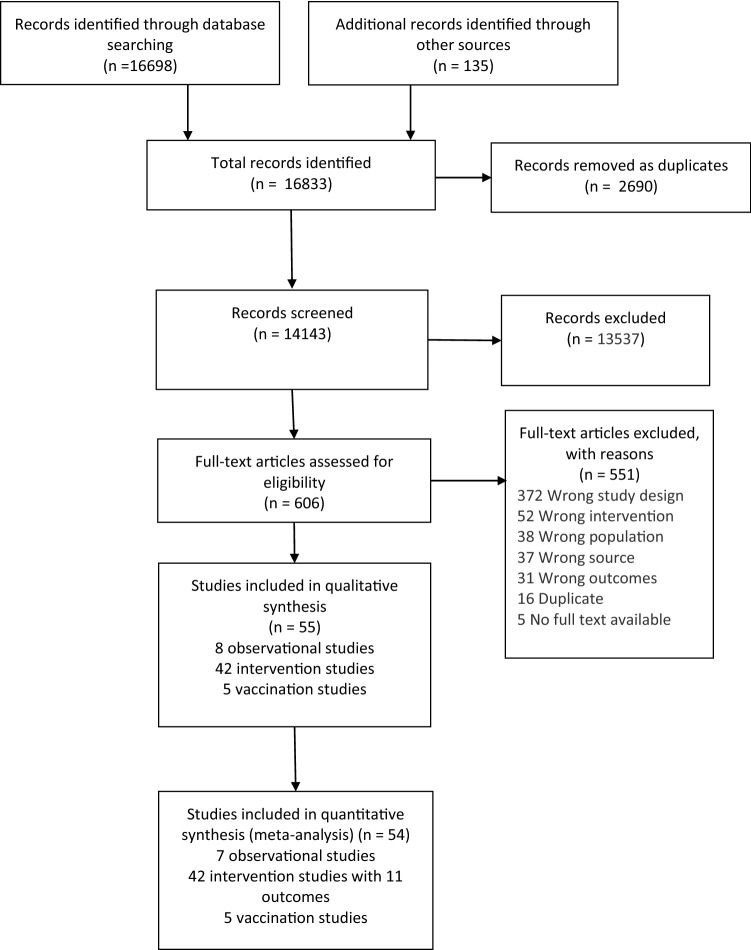
Preferred reporting items for systematic reviews and meta-analysis (PRISMA) flow diagram

The systematic search information flow is depicted in Fig. 1. The primary search returned 16,698 records, and an additional 135 were added through secondary manual searches. After screening, 606 full texts were read and assessed for inclusion. A total of 551 articles were excluded mainly because of the study design not meeting the inclusion criteria. After eligibility assessment 55 studies consisting of seven observational prospective studies and 48 RCTs (including six studies focussed on vaccination) met the inclusion criteria. The characteristics of these studies are given in ESM Tables S2–S4.

### Risk of Bias

We classified six observational studies as being at moderate risk of bias and one at high risk because they used the self-reported method to measure physical activity, contained limited adjustment for important covariates, lacked sensitivity analyses (e.g., to explore reverse causation) and did not include reporting of missing data (ESM Table S5). We judged 20 RCTs as being at moderate risk of bias and 12 at high risk because of poor randomisation, selection bias and poor reporting and management of missing data. The remaining 10 were at low risk (ESM Table S6). For vaccination studies, two were low risk while two were at moderate risk of bias and two at high risk because of selection bias and overall design issues. Details of quality assessment in each domain are given in ESM (Tables S5, S6 and S7).

### Synthesis

#### Observational Studies

**Fig. 2:**
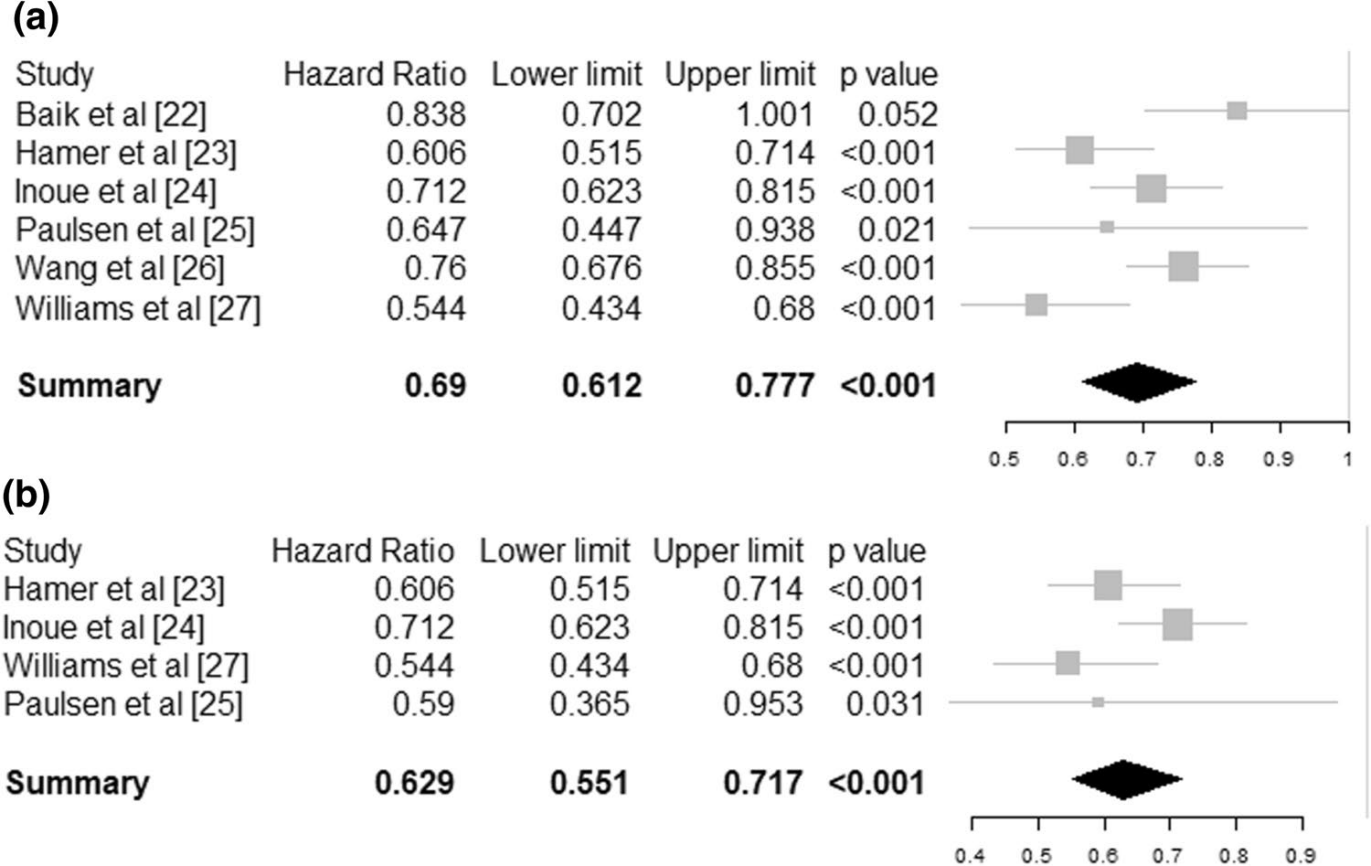
Forest plot for observational studies. **a** Risk of community-acquired infection, **b** risk of infectious disease mortality. Size of the square represent the weight of each study in the meta-analysis

Six observational studies could be meta-analysed [22–28]. Neuman et al. [28] and Baik et al. [22] reported on the same cohort and we included only Baik et al. [22] in the meta-analysis because it had a larger sample size from combining two cohorts. The pooled effect showed a statistically significant 31% risk reduction (hazard ratio [HR] 0.69 95% CI [0.61–0.78], *I*^2^ = 20.4%) for community-acquired infectious disease for people engaging in levels of regular physical activity equal or over the recommended 150 min per week compared to those below that level (Fig. 2a) based on a total sample of *N* = 557,487 individuals. The risk of infectious disease mortality (mostly pneumonia) was reduced by 37% (HR 0.64 95% CI [0.59–0.70], *I*^2^ = 40.0%) for individuals who met the recommended physical activity guidelines compared to individuals who did not meet the guidelines (Fig. 2b) based on a total sample of *N* = 422,813 individuals.

#### Physical Activity and Laboratory‐Assessed Immune Parameters

##### White Blood Cell Count (Leukocytes)

Ten studies (*N* = 384 individuals) [29–38] reported on total white blood cell count after a physical activity intervention programme involving 15–120 min (median 30 min) of moderate to vigorous intensity aerobic training (*n* = 7 studies, walking, running or cycling) or a combination of aerobic and resistance training (*n* = 3 studies) delivered 3–5 times per week and lasting from 4 to 26 weeks (median 12 weeks). There was no effect of physical activity interventions compared to control with a pooled overall lower white blood cell count of 519 cells/µL (95% CI [− 11 to 1049], *p* = 0.055) (ESM Figure S1). The heterogeneity between studies was moderate, *I*^2^ = 47.9%.

##### Innate Immune System Cell Counts

In total, 16 studies [29, 30, 32, 34–37, 39–47] reported on innate immune system cell count after a physical activity intervention programme involving 15–120 min (median 45 min) of moderate to vigorous intensity aerobic training (*n* = 4 studies, walking, running or cycling) [39–41, 46] or a combination of aerobic and resistance training (*n* = 2 studies) [35, 43] delivered 1–5 times per week (median 3) and lasting from 4 to 26 weeks (median 12 weeks). There was a statistically significant effect of physical activity interventions (ESM Figure S2) compared to control with a pooled lower neutrophil count of 704 cells/µL (95% CI [− 1340 to − 68], *p* = 0.030, *I*^2^ = 50.2%) for *N* = 305 individuals, but not for monocytes (MD = 18 cells/µL 95% CI [− 18 to 54], *p* = 0.325, *I*^2^ = 50.2%, *N* = 185 individuals) or NK cells (MD = − 15 cells/µL 95% CI [− 47 to 18], *p* = 0.378, *I*^2^ = 46.5%, *N* = 461 individuals).

##### Adaptive Immune System

*Total lymphocytes* Two studies investigated the effect of resistance training [48, 49], three combined resistance and aerobic (walking, cycling) physical activity [31, 32, 35] and all other studies used aerobic physical activity intervention only. Training programmes involved moderate to vigorous intensity activities for a minimum of 30 min twice a week and lasted between 4 and 26 weeks (median 8 weeks). There was a statistically significant effect of physical activity intervention compared to control with a pooled lower total lymphocyte count of – 244 cells/µL (95% CI [− 475 to 13], *p* = 0.038) for healthy adults, but not in other groups or overall (MD = − 60 cells/µL, 95% CI [− 277 to 157], *p* = 0.589, *I*^2^ = 41.8%, *N* = 498 individuals) (ESM Figure S3).

**Fig. 3:**
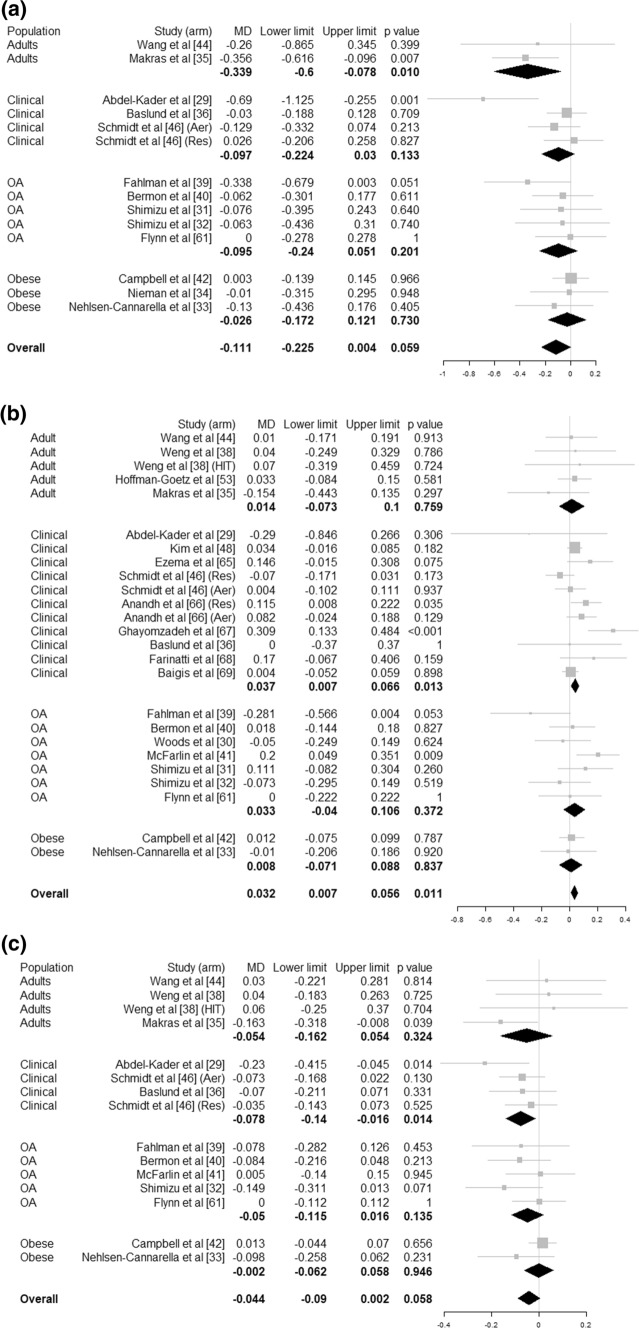
Forest plot for T cell counts: **a** CD3+, **b** CD4+ and **c** CD8+ for healthy adults, clinical groups, obese adults and older adults (OA). Mean difference (MD) is in cell/pL. Size of the square represent the weight of each study in the meta-analysis

*T cells *(*CD3+*) Training in these studies had a median frequency of 5 sessions per week, median duration of 40 min, lasting between 1 and 26 weeks (median 10 weeks) and involved aerobic activity (*n* = 7), resistance training (*n* = 5) and combined aerobic and resistance interventions (*n* = 4). There was no statistically significant effect of physical activity intervention (MD = − 111 cells/µL (95% CI [− 225 to 4], *p* = 0.059, *I*^2^ = 26.8%, *N* = 751 individuals) (Fig. 3a).

*T cell helpers *(*CD4+*) Training in these 24 studies had a median frequency of 3 sessions per week, median duration of 40 min, lasting between 1 and 26 weeks (median 10 weeks) and involved aerobic activity (*n* = 10), resistance training (*n* = 9) and combined aerobic and resistance activity (*n* = 6) at light to vigorous intensity. The meta-analysis (Fig. 3b) showed a statistically significant effect of physical activity intervention compared to control with a pooled higher T cell (CD4+) count of 32 cells/µL (95% CI [7–56], *p* = 0.011, *I*^2^ = 33.0%, *N* = 1112 individuals) overall and the same for clinical populations (MD = 37 cells/µL (95% CI [7–66], *p* = 0.013).

*T cells cytotoxic *(*CD8+*) Median characteristics for the studies reporting on the CD8+ lymphocytes sub-population were 3 sessions/week of 40 min for 8 weeks and involved resistance training (*n* = 8), aerobic activity (*n* = 9) or a combination of both (*n* = 3). The meta-analysis (which excluded seven studies which reported in different units [30, 46, 48, 50–53]) showed no significant effect of physical activity interventions compared to control for CD8+ count (MD = − 44 cells/µL, 95% CI [− 90 to 2], *p* = 0.058, *I*^2^ = 8.1%, *N* = 896 individuals). However, there was a statistically significant difference for clinical populations with lower CD8+ count of – 78 cells/µL (95% CI [− 140 to − 16], *p* = 0.014) (Fig. 3c).

##### Immunoglobulin

**Fig. 4:**
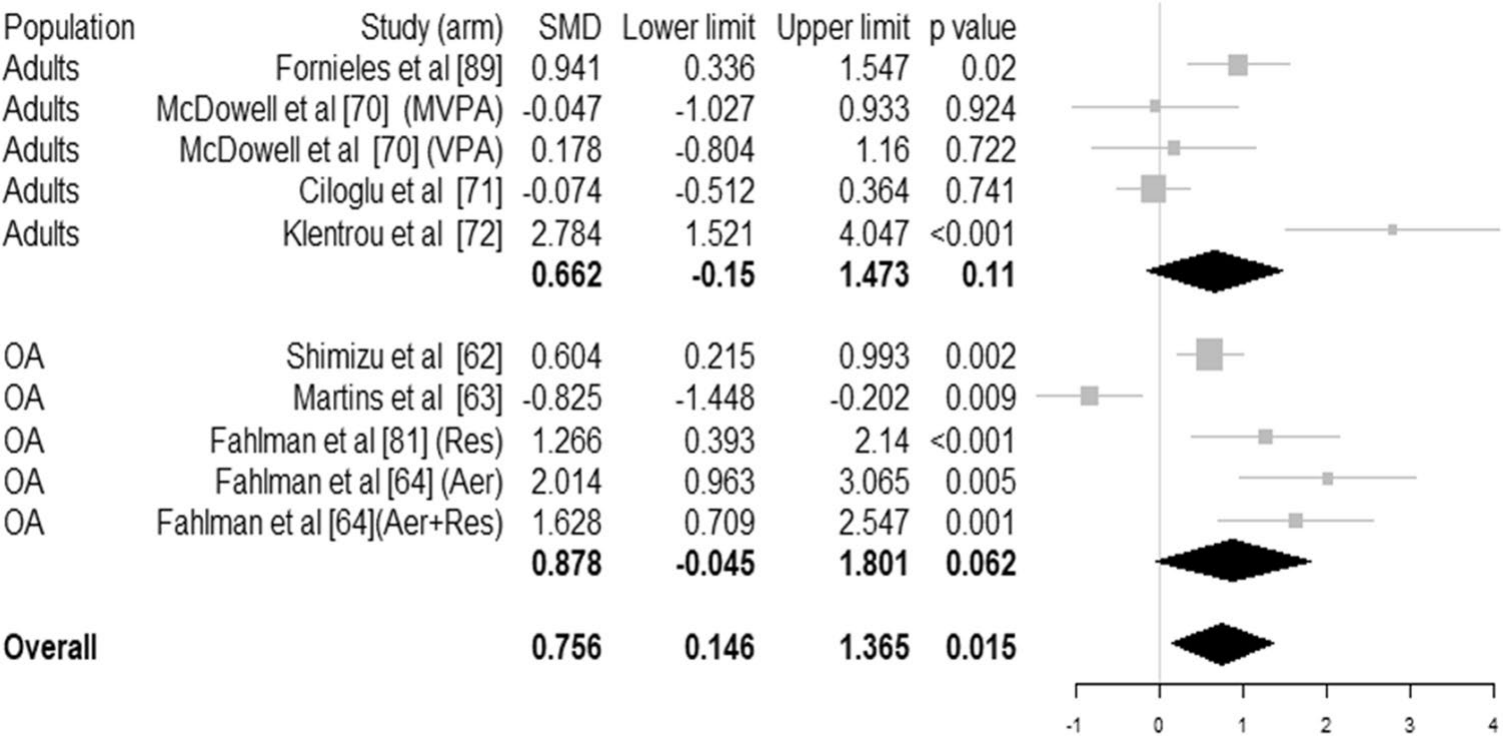
Forest plot for immunoglobulin concentration of salivary IgA (SIgA) for healthy adults and older adults (OA). Size of the square represents the weight of each study in the meta-analysis. For studies with several arms, arms are indicated. SMD represented the standardised mean difference

Our meta-analyses (Fig. 4) showed a statistically significant effect of physical activity interventions (median characteristics: 3 times per week, moderate to vigorous intensity, 30 min in length for 15 weeks) on salivary IgA concentration overall (SMD 0.756 95% CI [0.146–1.365], *p* < 0.015, *I*^2^ = 84.3%, *N* = 435 individuals). No statistically significant effect of physical activity was detected for serum IgA, IgG or IgM (ESM Figure S4).

#### Vaccination Studies

**Fig. 5:**
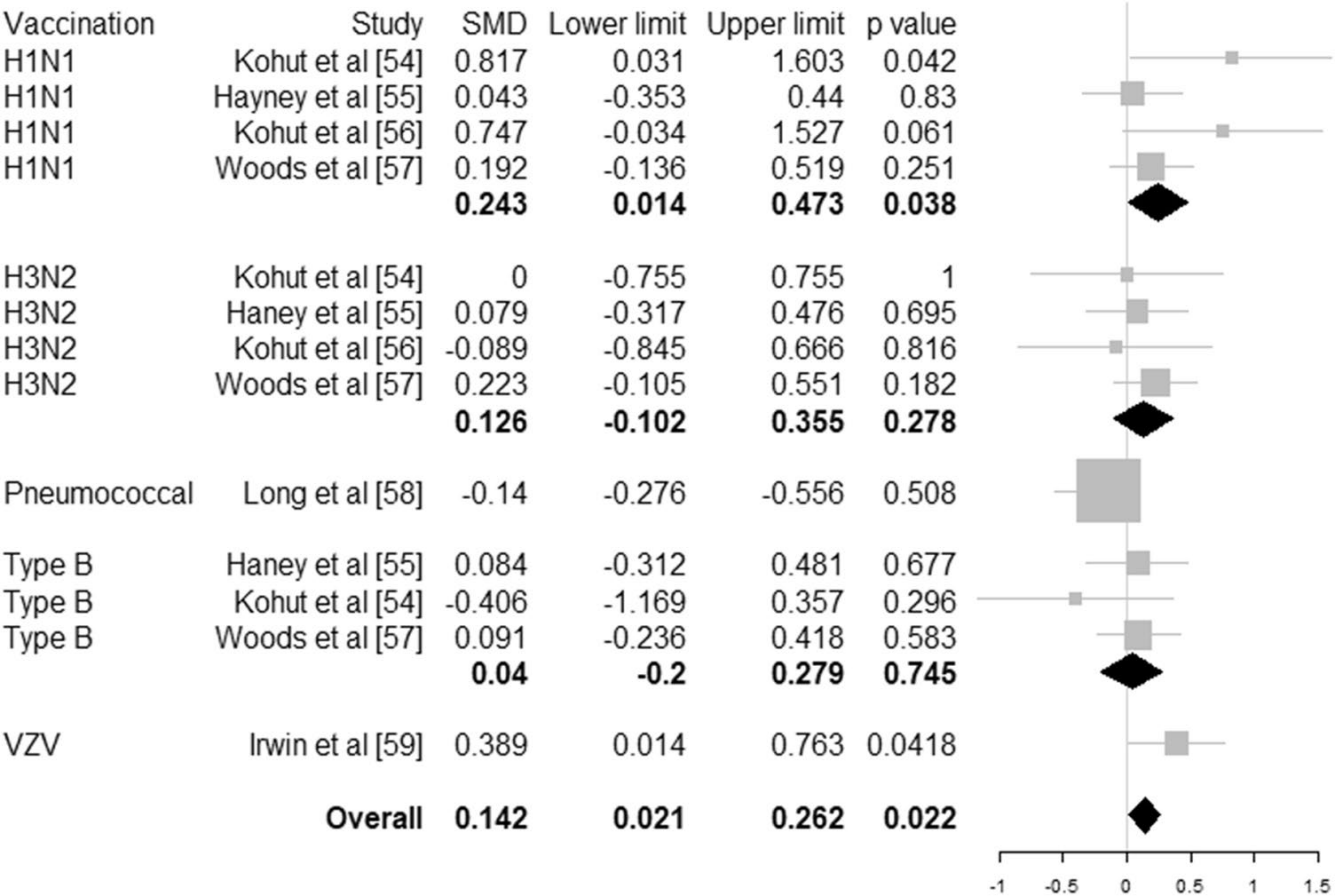
Forest plot for antibodies titres after vaccination per vaccination type. Size of the square represents the weight of each study in the meta-analysis. SMD represented the standardised mean difference

Six studies (*N* = 497 individuals) investigated the effect of physical activity interventions on the outcomes of vaccination and reported differences in antibody titres for H1N1, H3N2, influenza type B [54–57], pneumococcal [58] and varicella zoster virus [59]. The median characteristics of the training programme were 3 sessions per week of 60 min for 20 weeks prior to vaccination involving aerobic or combined aerobic and strengthening exercises [55]. Pooled effects showed a statistically significant effect of physical activity compared to control with higher antibody titres (SMD = 0.142 95% CI [0.021–0.262], *p* = 0.022, *I*^2^ = 0.00%) (Fig. 5).

### Heterogeneity, Publication Bias, Sensitivity Analysis and Influence of Intervention Characteristics

We observed no discernible signs of publication bias (ESM Figures S5 to S15) for any of the outcomes considered. Heterogeneity was low to moderate for most outcomes but high for salivary IgA. Sensitivity analysis, taking into account the quality of the studies, did not change the results significantly. Meta-regression did not show statistically significant trends or moderating effects of physical activity intervention characteristics (frequency, intensity, time, type and duration) or sub-population group for any of the outcomes. We could not identify a specific source of heterogeneity. None of the intervention characteristics or population subgroups explain observed heterogeneities. The heterogeneity is likely to be due to a combination of factors including the measurement methods, heterogeneity in the samples, and compliance with physical activity programmes.

## Discussion

Previous reviews and meta-analyses of the role of physical activity in immunity and risk of infectious disease were based on the risk of self-reported upper respiratory tract infection and proved inconclusive due to a low level of evidence and the very small number of published studies [13, 14]. In contrast, this meta-analysis which focuses on objective markers and includes a larger number of studies provides some clear and consistent results. This meta-analysis shows that higher levels of habitual physical activity are associated with a 31% lower prospective risk of infectious disease and 37% lower risk of infectious disease-related mortality.

We found evidence of significant changes in specific immune parameters as a result of regular physical activity. The physical activity interventions, lasting on average a median 12 weeks and including aerobic (walking, running, cycling) or resistance or combined aerobic and resistance activity delivered 3–5 times per week for an average of 30 min at moderate to vigorous intensity, resulted overall in higher concentrations of CD4 T cell helpers and salivary immunoglobulin IgA, and a lower concentration of neutrophils.

While the full role of CD4 T cells is not fully understood, they are thought to carry out a wide variety of functions within the immune system [73]. CD4 T cells are orchestrators, regulators and direct effectors of the immune response. CD4 T cells play a role in immunosurveillance, continuously monitoring for pathogens. They induce an early inflammatory response that contributes to a rapid and more robust immune response but also regulate this response and chronic inflammation. They enhance the response and memory of other immune cells but also are a direct effector mediating pathogen clearance [73, 74]. The elevated concentration of CD4 T cells found in this meta-analysis suggests that regular physical activity strengthens these functions within the immune system and results in a faster response.

The primary function of SIgA is to protect the mucosal surface against invasion by pathogens [75]. It can be regarded as the first line of defence of the immune system against external pathogens [76]. In addition, SIgA plays a variety of other roles and in particular, it acts as an anti-inflammatory, down-regulating inflammation processes [77]. The higher concentration of SIgA shown by our meta-analysis indicates that regular physical activity also strengthens the mucosal barrier to pathogens and the body's first line of defence.

Neutrophils are the most abundant white blood cells, are considered as the main effector of pathogen clearance and are the first white blood cells recruited upon infection. The observed decrease in neutrophil cell count associated with regular physical activity might, therefore, be interpreted in isolation as a sign that physical activity might depress the immune system. However, we did not observe a decrease in total white cell counts associated with regular physical activity and neutrophils play other roles within the immune system [78]. In particular, neutrophils are involved in chronic inflammation and an elevated neutrophil count is often regarded as a marker of chronic inflammation. It is well established that regular physical activity is associated with reduced levels of chronic inflammation and concentrations of inflammation markers [79, 80]. Therefore, an alternative interpretation of the observed decreased level of neutrophils is that it is a consequence of the effect of regular physical activity on chronic inflammation. This is congruent with the observed increased concentrations of SIgA and CD4 as this might also increase their anti-inflammatory action. It is also consistent with a report on the effect of acute exercise [12].

Taken together there is evidence that regular moderate to vigorous physical activity might contribute to a more effective immune system and response providing enhanced protective immunity to infections. The strength of the immune system and its efficacy cannot be fully ascertained by immune cell count and antibody concentration alone. However, our results for laboratory‐assessed immune parameters are consistent with the observed significant reduction in risk of community-acquired infection in this meta-analysis and some previous reports on decreased risk of self-reported upper-respiratory tract infections [5, 81, 82]. Taken together it is logical to think there is evidence that regular moderate to vigorous physical activity might contribute to a more effective immune system and response providing enhanced protective immunity to infections.

This suggests that people should be encouraged to engage in regular physical activity to strengthen their immune system, and decrease their risk of infectious disease and mortality related to infectious disease. Future public health guidelines for physical activity should consider integrating evidence about the impact of physical activity on infectious disease as well as chronic diseases and make recommendations.

The current evidence base does not provide enough information to be very specific about how time, frequency, duration and type of physical activity influence the effect on immune defence to infectious disease. However, the level of physical activity recommended (150 min per week of moderate to vigorous physical activity combining aerobic and strengthening activity) by current guidelines [83] for prevention of chronic disease appears to be also protective against infectious disease and infectious disease mortality. It is likely that a dose–response relationship exists. This has been hypothesised previously [5, 12] based on epidemiological studies of incidence of self-reported upper respiratory tract infection epidemiological studies. It is thought that this relationship has a J-shape with increased risk of infection with heavy exertion. The activity level associated with increased risk remains unclear. We found no evidence of detrimental effect of physical activity with increased volume or intensity in the general population and up to moderate to vigorous intensity and volume ranging from 60 to 600 min/week. This suggests that within the general population, engaging in regular physical activity at any intensity up to moderate to vigorous activity is safe with respect to risk of infection. Consequently, future public health guidelines should integrate the protective benefit of physical activity against infectious diseases. This might help promote physical activity more effectively for prevention and management of outbreaks, epidemics and pandemics.

In addition, physical activity programmes could be used as an adjunct to vaccination campaigns because while a meta-analysis on the effect of acute exercise intervention on influenza immunisation was inconclusive [84], the present meta-analysis demonstrates that regular physical activity significantly increases antibody levels after vaccination, including in older adults. This is consistent with the conclusion of a narrative review of the effect of chronic exercise on immunisation [85]. The data suggest that regular physical activity programmes involving around 150 min per week of moderate to vigorous physical activity for 20 weeks prior to vaccination could be necessary.

The mechanism by which physical activity influences the immune system remains unclear and a matter of debate, but is likely to involve multiple pathways, both physiological and psychological [5, 86]. Physical activity is also well known to improve chronic conditions such as diabetes or obesity that increase the risk of severe complications and mortality due to infectious disease. In addition, physical activity is effective for stress management which in turn affects both the immune system and inflammatory responses [87].

### Risk of Bias and Future Research

While the evidence base was overall of good quality with most studies being of moderate to low risk of bias, future research should seek to improve certain points. For observational studies, better reporting and treatment of confounding factors would improve the situation. In addition, the main limitation within observational studies was that physical activity was self-reported rather than objectively measured. Self-reported measures of physical activity tend to lead to attenuation of associations [88]. Future studies should preferably use device based measures of physical activity. For RCTs, better reporting of randomisation would improve risk of selection bias, and standardised reporting of between group differences at the end of the trial rather than focussing on within group changes would be better practice. Longer follow-up time would also indicate the time scale involved and if the effects are lasting.

Finally, it is likely that the response to regular physical activity will vary between different vaccinations and in different populations. The current body of evidence is too small currently to draw specific conclusions. Further studies are warranted to understand how physical activity programmes could be tailored for specific vaccinations and populations.

### Strengths and Limitations

The strengths of our meta-analysis included: the wide-ranging systematic search strategy; the focus on robust evidence obtained from RCTs and prospective studies only; a thorough check for duplicate data; the comprehensive quality assessment of the primary studies; the extensive range of outcome analysed; and the focus on objectively measured outcomes. The main limitation is that we had to combine heterogeneous information without being able to pinpoint precisely the source of heterogeneity. The meta-analysis of laboratory immune parameters focused on cell counts and antibody concentration and this does not allow a full understanding of the link between changes in the immune system and the decrease in risk of infectious disease associated with regular physical activity. Finally, the evidence base is not large enough currently to be able to understand more precisely the impact of time, duration, frequency and intensity of physical activity in different populations and for different infectious diseases and vaccinations.

## Conclusion

The results from this systematic review and meta-analysis reveal that regular physical activity increases resistance to infectious disease in the general population. Regular physical activity should be promoted in the general population to decrease the risk of community-acquired infection and infectious disease mortality, strengthen the potency of immunisation programmes and help lessen the impact of pandemics such as the recent COVID-19.

## Supplementary Information

Below is the link to the electronic supplementary material.

Supplementary file1 (DOCX 5865 kb)

## Declarations

### Availability of data and material

The datasets generated during and/or analysed during the current review are available from the corresponding author on reasonable request.
