# Supplementary material for: Effects of Regular Physical Activity on the Immune System, Vaccination and Risk of Community-Acquired Infectious Disease in the General Population: Systematic Review and Meta-Analysis
Source: Sports Med. 2021 Apr 20;51(8):1673–86. doi: 10.1007/s40279-021-01466-1 (PMC8056368; doi:10.1007/s40279-021-01466-1)
Supplement: Supplementary file 1 — Supplementary file1 (DOCX 5865 kb) [file 40279_2021_1466_MOESM1_ESM.docx]

**Electronic Supplementary Material**

**Title:** Effects of regular physical activity on the immune system, vaccination and risk of community acquired infectious disease in the general population: Systematic review and meta-analysis.

Table S1. Example search strategy PubMed

| 1 | exp fitness/ or exp physical fitness/ or exp training / or exp resistance training/ or exp aerobic training/ or exp combined training/ yoga, Tai chi/ |
| --- | --- |
| 2 | (exercise adj3 or fitness adj3).ti.ab |
| 3 | exercise.mp. or exp Exercise/ |
| 4 | (physical adj3 activ$).ti,ab |
| 5 | 1 or 2 or 3 or 4 |
| 6 | exp infectious disease/ or exp communicable disease/ or exp respiratory infections/ or exp COVID-19/ or exp corona virus/ or exp pneumonia/ or exp immune system/or exp immune function/or exp immune cell/ |
| 7 | (corona adj3).tw. |
| 8 | (infect$ or diseas$ or virus or corona adj3)).ti,ab |
| 9 | viral infection.mp. or exp viral infection/ |
| 10 | lower respiratory tract infection.mp or exp lower respiratory tract infection/ |
| 11 | 6 or 7 or 8 or 9 or 10 |
| 12 | exp killer cell/ or exp t?cell/ or exp leukocyte/ or exp lymphocytes/ or exp adhesion molecules/ or exp white blood cell count/ or exp neutrophils/ or exp monocytes/ or expp kill cells/ or exp natural killer cells/ or exp/ or exp immunoglobulins/ |
| 13 | (immunoglobin$ or immun$) adj3)).ti,ab |
| 14 | antibod$)adj3 |
| 15 | (vaccin$)adj3 |
| 16 | 12 or 13 or 14 or 15 |
| 17 | exp infectious disease mortality/ or exp all?cause mortality/ |
| 18 | exp death/ |
| 19 | 17 or 18 |
| 20 | 11 or 16 or 19 |
| 21 | exp adult/ or exp human/ |
| 22 | adult$ or adult adj3).ti.ab |
| 23 | 21 or 22 |
| 21 | 5 and 20 and 23 |

**Studies Characteristics**

Table S2. Descriptive characteristics of the 7 observational studies included.

| **Study (Year)** | **Design and Population** | **Physical activity (PA) assessment method** | **Outcome / Follow Up** | **Main finding/Conclusions** | **Risk of Bias** |
| --- | --- | --- | --- | --- | --- |
| Baik et al. (2000)[1] | Prospective  **Population:**  N= 104,491 (Male 26429)  Age: 27-79y    **Study/cohort name:**  Health Professional Follow-up Study + Nurse's Health Study | **Tool:**  Self-reported 8 questions  **PA Units:**  MET-hours | **Outcome:**  Community acquired pneumonia  **Follow Up:**  6 years for male, 2 years for female | PA was inversely associated with risk of community acquired pneumonia  only among women. | Moderate |
|  |  |  |  |  |  |
| Hamer et al. (2019)[2] | Prospective  **Population:**  N= 97,844  Age: 47.1±17.1y  **Study/cohort name:**  Health Survey England/Scottish Health Survey | **Tool:**  Self-reported freq/duration in last 4 weeks across different domains (validated against accelerometery).  **PA Units:**  Meeting PA guideline and MET-hours | **Outcome:**  infectious disease mortality/ pneumonia mortality  **Follow Up:**  9.4 years | PA inversely associated with infectious disease mortality (not pneumonia). | Moderate |
|  |  |  |  |  |  |
| Paulsen et al. (2017)[3] | Prospective  **Population:**  N= 64,027  Age: 34-63y    **Study/cohort name:**  Norwegian HUNT | **Tool:**  Self-reported PA/ intensity in past year.  **PA Units:**  Categories:   - None - Slight: <3h/wk light PA - Moderate: >3h/wk light PA and < 1h/wk vigorous - High: > 1h/wk vigorous PA | **Outcome:**  Blood stream infection detected in blood culture; death within 30 days of infection detected  **Follow Up:**  14.8y | PA inversely associated with blood stream infection mortality (not pneumonia). | Moderate |
|  |  |  |  |  |  |
| Wang et al. (2014)[4] | Prospective  **Population:**  N= 30,183  Community dwelling adults Age: >45y    **Study/cohort name:**  Reasons for Geographic and Racial Differences in Stroke (REGARDS) study, a national, population-based | **Tool:**  Phone interview, weekly frequency exercise (intense enough to work up a sweat).  **Physical activity Units:**  *PA frequency:*   - None - Slight: 1-3/wk; - 4+ /wk | **Outcome:**  *International consensus definitions:*  Community acquired sepsis as hospital treatment for an infection with two or more systemic inflammatory response syndrome criteria  **Follow Up:**  9y | Inactivity associated with increased long-term rates of community-acquired sepsis. | Moderate |
|  |  |  |  |  |  |
| Inoue et al. (2007)[5] | Prospective  **Population:**  N= 110,792  Community based adults  Age: 40-79y  **Study/cohort name:**  Japan collaborative cohort study | **Tool:**  Self-reported hr/wk playing sports and walking  **Physical activity Units:**   - Sports: <1, 1-2, 3-4, >4 hr /week - Walking: <0.5, 0.5, 0.5-1, >1 hr/day | **Outcome:**  Pneumonia death  **Follow Up:**  15y | Walking associated with lower risk of pneumonia mortality. | Moderate |
|  |  |  |  |  |  |
| Williams et al. (2014)[6] | Prospective  **Population:**  N= 109,352 runners and 40,798 walkers  Age: 40-61y    **Study/cohort name:**  The National Walkers' and Runners' Health Studies | **Tool:**  Self-reported running and walking (METS)  **Physical activity Units:**  MET-hours | **Outcome:**  Pneumonia death  **Follow Up:**  17y | Higher doses of running and walking were associated with lower risk of pneumonia mortality in a dose-dependent manner, and the effects of running and walking appear equivalent. | High |
|  |  |  |  |  |  |
|  |  |  |  |  |  |
| Neuman et al. (2010)[7] | Prospective  **Population:**  N= 83,165 Women  Age: 27-44y (baseline)  **Study/cohort name:**  Nurse Health Study NHS (Note re | **Tool:**  Self-reported 8 question  **Physical activity Units:**  MET-hours | **Outcome:**  Community acquired pneumonia  **Follow Up:**  12y | Women in the highest quintile of physical activity were less likely to develop pneumonia than women in the lowest quintile. | Moderate |

Table S3. Descriptive characteristics of the 42 experimental physical activity programme studies included.

| **Study (Year)** | **Design and Sample** | **Exercise/PA and control conditions** | **Intervention Parameters (F.I.T.T.)** | **Main finding/Conclusions** | **Risk of Bias** |
| --- | --- | --- | --- | --- | --- |
| Wang et al. (2011)[8] | RCT  N= 60 (12 each group)  Healthy sedentary males  **Group allocation:**  *(mean age +/- SEM)*  - Hypoxic absolute exercise(H-AE): 23.3±0.7y  -Hypoxic relative exercise (H-RE): 24.4±0.4y  - Normoxic exercise (N-E): 23.1±0.8y  - Hypoxic control (H-C): 22.9±0.7y  - Hypoxic control (H-C): 22.9±0.7y  - Normoxic control (N-C): 23.4±0.9y | **Exercise:**  Bicycle ergometer (Corvial 400; Lode) for N-E, H-RE and H-AE.  **Control:**  normoxic (21% O2) control (N-C)  hypoxic (15% O2) control (N-C) | **Frequency:**  5d/week  **Intensity:**  50% maximal work rate  **Time:**  30 min  **Type of Exercise:**  Aerobic training  **Length:**  4 weeks | A 15% O2 exercise training reduces terminally differentiated NK subsets and up-regulates the expression of activating molecules and cytotoxic granule proteins in NKs. | High |
|  |  |  |  |  |  |
| Hoffman-Goetz et al. (1990) [9] | RCT (matched on maximal O2 uptake)  N= 18 (9 each group)  Healthy young males  **Group allocation:**  *(mean age +/- SEM)*  - Exercise group: 24.5±0.9y  - Control group: 22.8±1.2y | **Exercise:**  Bicycle ergometer.  **Control:**  Resting during the exercise sessions | **Frequency:**  5 days  **Intensity:**  65% VO2max  **Time:**  60 min  **Type of Exercise:**  Aerobic training  **Length:**  5 days | Repeated exposure to submaximal exercise results in consistent increases in the percentage of NK cells, demonstrating that the exercise effects on T-lymphocyte subset percents were variable over time. | High |
|  |  |  |  |  |  |
| Ciloglu et al. (2005)[10] | RCT  N= 90 (30 each group)  Sedentary women  (45-65y)  **Group allocation:**  *(mean age +/- SD)*  -Outdoor exercise:55.0±3.5y  - Indoor exercise:54.6±2.1y  -Sedentary control:54.9±1.8y | **Exercise:**  Walking track or treadmill  **Control:**  Telephone calls | **Frequency:**  5 d/week  **Intensity:**  60% MHR  **Time:**  30 min  **Type of Exercise:**  Aerobic training  **Length:**  12 weeks | Moderate intensity aerobic exercise is  associated with fewer episodes of URTI in healthy postmenopausal Turkish women, but this does not seem to be related to salivary IgA concentrations. | Moderate |
|  |  |  |  |  |  |
| Unal et al. (2005)[11] | RCT  N= 24 (12 each group)  Sedentary male university students and officers  **Group allocation:**  *(mean age +/- SD)*  - Aerobic Group: 25.67±3.79y  - Anaerobic Group: 24.83±2.89y | **Exercise:**  Bicycle ergometer  **Comparing Group:**  Bicycle ergometer | **Frequency:**  3 d/week  **Intensity:**  10% below/above threshold (HR)  **Time:**  30 min  **Type of Exercise:**  Aerobic vs anaerobic trainings  **Length:**  8 weeks | Cytolytic activity of blood increases further after short-term anaerobic exercises.  The effects of anaerobic exercises versus aerobic on leukocyte and lymphocyte seem to be much more obvious. | Moderate |
|  |  |  |  |  |  |
| Flynn et al. (1999)[12] | RCT  N= 29 (14 control group)  Elderly women (67-84y)  **Group allocation:**  *(mean age +/- SD)*  - Resistance exercise: 72.6±3.5 y  -Control group: 72.9±4.9y | **Exercise:**  Resistance exercise training  *(leg extension, leg curl, plantar flexion, and dorsiflexion; leg*  *abduction, leg adduction, hip extension, and hip flexion)*  **Control Group:**  1-wk period of acclimation to resistance training and after inactive | **Frequency:**  3 d/week  **Intensity:**  3x8 rep at 70%/80% 1RM 1st and 2snd weeks  **Time:**  NP  **Type of Exercise:**  Resistance training  **Length:**  10 weeks | Resistance training did not influence resting immune measures in women aged 67–84 yr. | Moderate |
|  |  |  |  |  |  |
| Nieman et al. (1998)[13] | RCT  N= 91 (14 control group)  Obese women (45.6 ± 1.1y)  **Group allocation (N):**  - Exercise group (21)  - Diet group (26)  - Exercise + Diet group (22)  - Control group (22) | **Exercise:**  Brisk walking  **Control Group:**  No exercise | **Frequency:**  5 d/week  **Intensity:**  60%-80% MHR  **Time:**  45 min  **Type of Exercise:**  Aerobic training  **Length:** 12 weeks | Moderate weight loss by energy restriction alone or a combination of exercise training and energy restriction, resulted in a decrease of MSLP, and no significant effect on NKCA, and monocyte/granulocyte phagocytosis and oxidative burst activity. | Low |
|  |  |  |  |  |  |
| Wang et al. (2011)[14] | RCT  N= 60 (30 each group)   Female college students  (19.3±1.8y)  **Group allocation:**  *(mean age +/- SD)*  - Exercise TAI CHI CHUAN (TCC): 19.5±2.1y  - Control group: 19.2±1.3y | **Exercise:**  TCC uses “meditation through movement” incorporating elements of balance, postural alignment, and concentration.  **Control Group:**  Normal activities | **Frequency:**  5d/week  **Intensity:**  NP  **Time:**  45 min  **Type of Exercise:**  Balance and meditation training  **Length:**  12 weeks | Regular long-term TCC practice might be a potential method to improve the cellular immune function (anti-virus and anti-infection) of people who lack physical exercise. | Moderate |
|  |  |  |  |  |  |
| Hutnick et al. (2005)[15] | Controlled (non-randomized) clinical trials (CCTs)  N= 49 (21 control group)  Patients with breast cancer  **Group allocation:**  *(mean age +/- SD)(range)*  -Exercise Group: 48.5±10.6y (29-69y)  - Control group: 52.3±9.2y (38-71y) | **Exercise:**  4 upper and lower body strength exercises  **Control Group:**  Normal life and cancer treatment | **Frequency:**  3d/week  **Intensity:**  Resistance: 8-12rep (1-3 set)  Aerobic: 60–75% functional capacity  **Time:**  40-90 min  **Type of Exercise:**  Resistance and aerobic training  **Length:**  6 months | Exercise may improve immune function by increasing lymphocyte activation in patients with breast cancer following treatment. | Moderate |
|  |  |  |  |  |  |
| Martins et al. (2009)[16] | RCT  N= 43 (21 control group)  Sedentary and independently living Older Men and Women (65-96 y)  **Group allocation:**  *(mean age +/- SD)*  -Exercise Group: 77.7±7.8y  -Control group: 73.2±4.5y | **Exercise:**  Low-impact rhythmic work sequences with music.  **Control Group:**  No exercise | **Frequency:**  3d/week  **Intensity:**  From 40 – 50 % HRR  To 71– 85 % HRR  **Time:**  45 min  **Type of Exercise:**  Aerobic training  **Length:**  16 weeks  (follow up 32 weeks) | Regular aerobic exercise may be effective in promoting IgA immunity and protecting against the deterioration in Sal-IgA without gender differences. | Moderate |
|  |  |  |  |  |  |
| Makras et al. (2005)[17] | RCT  N= 57 (9 control group)  Male recruits of the Hellenic Air force  **Group allocation:**  *(mean age +/- SD)*  - Exercise Group: 21.3 ± 3y  - Control group: 23.3 ± 3.4y | **Exercise:**  Military training intermittent physical exercise repeated exercise sessions of different types (running, walking, gymnastics, climbing, with/out backpacks, etc.)  **Control Group:** light work in the barracks | **Frequency:**  5d/week  **Intensity:** NP  **Time:**  15-120 min  **Type of Exercise:**  Military training  **Length:** 4 weeks | 4 weeks of military training consisting of intermittent moderate exercise resulted in a significant increase in CD4+ T-lymphocytes and reduction in neutrophils. | High |
|  |  |  |  |  |  |
| Klentrou et al. (2002)[18] | RCT  N= 20 (10 each group)  Sedentary adult men or women (25-50 yrs)  **Group allocation:**  - Active Group  -Control Group | **Exercise:**  Moderate exercise training  (stationary bicycles, treadmills, stair climbers, or combined/cross-training)  **Control Group:**  No exercise | **Frequency:**  3d/week  **Intensity:**  75%HRR  **Time:**  45 min (30 min aerobic+15 min  Stretching)  **Type of Exercise:**  Concurrent training  **Length:**  12 weeks | While the [IgAs] was significantly increased in the exercise group following the training period, the increase appeared to be only partially related to the overt symptoms of illness.  These findings suggest that regular moderate exercise may contribute to chronic immunosurveillance in sedentary individuals | Moderate |
|  |  |  |  |  |  |
| Shimizu et al. (2011)[19] | RCT  N= 24 (14 each group, 7 men)  Healthy, sedentary, elderly subjects  **Group allocation:**  *(mean age +/- SD)(range)*  - Exercise group: 67.1± 1.0y (61-76y)  - Control group: 67.56± 0.7y (62-79y) | **Exercise:**  Strength machines  **Control Group:**  Normal physical activity levels | **Frequency:**  5d/week (3 at home)  **Intensity:**  20-40% 1RM  **Time:** NP  **Type of Exercise:**  Resistance training  **Length:**  12 weeks | Strength training in elderly people is associated with increased CD28-expressing Tc cells and CD80-expressing monocytes. | High |
|  |  |  |  |  |  |
| Mitchell et al. (1996)[20] | RCT  N= 21 (10 control group)  College-aged males  **Group allocation:**  *(mean age +/- SD)*  - Exercise group: 23.4± 7.0y  - Control group: 20.1± 1.9y | **Exercise:**  Cycle Ergometer  **Control Group:**  No exercise | **Frequency:**  3d/week  **Intensity:**  75% VO2max  **Time:**  30 min  **Type of Exercise:**  Aerobic training  **Length:**  12 weeks | Moderate endurance exercise did not alter resting immune function as determined by mitogen stimulated lymphocytes proliferation, total circulating lymphocytes, or Ig levels. | Moderate |
|  |  |  |  |  |  |
| Shimizu et al. (2007)[21] | RCT  N= 156 (33 control group, 62 males in total)  Independently living, sedentary elderly  **Group allocation:**  *(range)*  - Exercise group: 60-83y  - Control group: 62-81y  4 additional specific age groups each allocation:   - 60–69-y-old males - over 70-y-old males - 60–69-y-old females - over 70-y-old females | **Exercise:**  Cycle ergometer+7 strength exercises (push-up, squat, sit-up, back-extension, leg extension, hip-extension, and leg-curl)  **Control Group:**  No exercise | **Frequency:**  5d/week  **Intensity:**  80% work rate of DPBP 1-3set x 10 rep  **Time:**  30 min aerobic training  30 min approx. Resistance (3s concentric +3s eccentric)  **Type of Exercise:**  Concurrent training  **Length:**  24 weeks | Enhancement of mucosal immune function following regular moderate exercise training occurs in elderlies in their 60s and over 70 years, and in both, males and females. | Moderate |
|  |  |  |  |  |  |
| Abd El-Kader et al. (2018)[22] | RCT  N= 60 (30 each group)  Sickle cell anaemia patient (ranged 25-40y)  **Group allocation:**  *(mean age* ± *SD)*  - Exercise group:25.16±7.14y  - Control group: 24.54± 7.63y | **Exercise:**  Treadmill running/walking  **Control Group:**  No exercise | **Frequency:**  3d/week  **Intensity:**  60-70% MHR  **Time:**  30 min  **Type of Exercise:**  Aerobic training  **Length:**  12 weeks | Aerobic training improves inflammatory markers and immune system in patients with sickle cell anaemia in asymptomatic steady state. | High |
|  |  |  |  |  |  |
| Campbell et al. (2008)[23] | RCT (stratified by body mass index)  N= 115 (86 each group)  Postmenopausal women (aged 50-75y)  **Group allocation:**  *(mean age* ± *SD)*  - Exercise group:25.16±7.14y  - Control group: 24.54± 7.63y | **Exercise:**  Walking and bicycling  **Control Group:**  Stretching + relaxation 60 min/week | **Frequency:**  5d/week  **Intensity:**  MVPA  **Time:**  45 min  **Type of Exercise:**  Aerobic training  **Length:**  12 months | There is no effect of aerobic exercise on in vitro immune function, despite excellent retention, high adherence, and demonstrable efficacy of the exercise intervention. | Moderate |
|  |  |  |  |  |  |
| Kim et al. (2006)[24] | RCT (random permuted  block design, random number table)  N= 42 (21 each group)  Allogeneic bone marrow transplantation patients  **Group allocation:**  - Bed exercise group  - Control group | **Exercise:**  Concentration on lower  abdomen for 3 min; put left ankle on right knee for 3 min; put right ankle on left knee for 2 min; and bend both knees for 2 min.  **Control Group:**  Bed rest | **Frequency:**  7d/week  **Intensity:**  LIPA  **Time:**  30 min  **Type of Exercise:**  Mobility Training  **Length:**  6 weeks | Bed exercise could be an effective nursing intervention with patients undergoing bone marrow transplantation by increasing the lymphocyte count during hospitalization. | Low |
|  |  |  |  |  |  |
| Hwang et al. (2016)[25] | RCT  N= 12 (6 each group)  Older women (68.3±5.6)  **Group allocation:**  *(mean age* ± *SD)*  - Exercise group: 64.5± 5.6y  - Control group: 68.3± 2.8y | **Exercise:**  Pilates  **Control Group:**  No Exercise | **Frequency:**  3d/week  **Intensity:**  40%-60% MHR  **Time:**  50 min  **Type of Exercise:**  Mobility Training  (Pilates)  **Length:**  12 weeks | A moderate-intensity Pilates exercise program can increase salivary flow rate and S-IgA secretion in older women. | High |
|  |  |  |  |  |  |
| Wang et al. (2011)[26] | RCT  N= 50 (10 each group)  Sedentary male  (aged 23.5 ± 0.8y)  **Group allocation:**  *(mean age* ± *SD)*  -Hypoxic-absolute exercise: 23.5±0.8y  - Hypoxic relative exercis: 22.3 ±0.7y  -Normoxic exercise: 21.5±0.7y  -Hypoxic control: 22.1+±0.4y  -Normoxic control: 22.9±0.4y | **Exercise:**  Bicycle ergometer (Corvial  400, Lode, AN Groningen, Netherlands)  - 21% O2 in normoxic  - 15% O2 in Hypoxic  **Control Group:**  No Exercise | **Frequency:**  5d/week  **Intensity:**  50 %MHR  **Time:**  30 min  **Type of Exercise:**  Aerobic training  **Length:**  4 weeks | Aerobic exercise shows promise as a positive immunomodulator in older adults after immunisation. | Moderate |
|  |  |  |  |  |  |
| Weng et al. (2013)[27] | RCT  N= 30 (10 each group)  Healthy sedentary males  (aged 22.5±1)  **Group allocation:**  *(mean age* ± *SE)*  - HIIT Exercise group: 22.3±0.2y  - MICT Exercise group: 22.5±1.0y  - Control group: 22.4±0.9y | **Exercise:**  HIIT and MICT in bicycle ergometer  **Control Group:**  No Exercise | **Frequency:**  5d/week  **Intensity:**  HIIT = 80% VO2Max (5x3min; density=1)  MCT = 60% VO2 Max  **Time:**  30 min  **Type of Exercise:**  Aerobic Training  **Length:**  5 weeks | HIIT is superior to MCT for enhancing aerobic fitness.  Moreover, either HIT or MCT effectively depresses apoptosis and promotes autophagy in CD4 lymphocytes and is accompanied by increased interleukin-4/interferon-γ ratio and decreased peroxide production during hypoxic exercise. | Moderate |
|  |  |  |  |  |  |
| Ezema et al. (2014)[28] | RCT  N= 33 (16 Control group)  HIV individuals  (aged 22-63y)  **Group allocation:**  *(mean age* ± *SE)*  -Exercise group: 40.07±9.72y  -Control group:32.47±10.41y | **Exercise:**  Jogging  **Control Group:**  No Exercise | **Frequency:**  3d/week  **Intensity:**  2 weeks 60% HHR  6 weeks 79% HHR  **Time:**  2 weeks 45 min  6 weeks 60 min  **Type of Exercise:**  Aerobic training  **Length:**  8 weeks | Moderate intensity aerobic exercise is an effective complementary therapy in lowering blood pressure and increasing CD4 cell count in PLWHA. | High |
|  |  |  |  |  |  |
| Schmidt et al. (2018)[29] | RCT  N= 67 (21 and 20 resistance and endurance exercise groups)  Breast cancer patients  **Group allocation:**  *(mean age* ± *SE)*  -Resistance exercise:53±12.55y  -Endurance exercise:56±10.15y  -Control group: 54±11.19y | **Exercise:**  -Squat, chest press, leg curl, rowing, leg extension, upper arm curl, upper arm extensors, shoulder press, abdominal bench and lats pull down  - Indoor bike (Tomahawk, Indoor cycling group, Germany).  **Control Group:**  Usual Care | **Frequency:**  2d/week  **Intensity:**  20rep*50% 1RM  11-14 Borg scale (MVPA)  **Time:**  60 min  **Type of Exercise:**  Resistance and aerobic training  **Length:**  12 weeks | Chemotherapy led to a decrease in nearly all measured immune cells.  Resistance or endurance training did not suppress cellular immunity any further. | High |
|  |  |  |  |  |  |
| Fahlman et al. (2003)[30] | RCT  N= 49 (4 groups)  Older adults  77±5y (65 -92y)  **Group allocation:**  -Aerobic Exercise: (n=11)  -Strength Exercise: (n=15)  -Aerobic + Strength: (n=11)  -Control group: (n=10) | **Exercise:**  - Chair squats, hip flexion, hip extension, standing abduction, standing adduction, chest press, lateral shoulder raises, seated row, abdominal curl-ups, biceps curls, triceps extension, calf raises, toe raises)  - Walking  **Control Group:**  Usual Care | **Frequency:**  3d/week  **Intensity:**  - Strength: 2 set*12reps  - Aerobic: 11-16 borg scale.  **Time:**  35 min  **Type of Exercise:**  Aerobic, Strength and concurrent trainings  **Length:**  16 weeks | Moderate exercise has a positive effect on mucosal immunity as measured by s-Ig A in low-functioning elderly, suggesting a greater resistance to upper respiratory-tract infection with involvement in an exercise programme. | Moderate |
|  |  |  |  |  |  |
| Anandh et al. (2013)[31] | RCT  N= 36 (12 each group, 26 males)  People with HIV/AIDS  (42.21±5.9y)  **Group allocation:**  -Aerobic exercise  -Resistance exercise  -Control group | **Exercise:**  - Treadmill walking/Arm ergo meter/Elliptical training.  - Progressive Resistance Exercise of major muscles.  **Control Group:**  No exercise | **Frequency:**  3d/week  **Intensity:**  - 50%, 60% & 70% HR at 1st, 2nd & 3rd months respectively  - 10RM per wk  **Time:**  60 min  **Type of Exercise:**  Aerobic and resistance trainings  **Length:**  12 weeks | Aerobic training and progressive resistance training can be recommended with confidence for improving functional capacity, immune system and quality of life and also a better adjunct to pharmacological therapy for people living with HIV/AIDS. | Low |
|  |  |  |  |  |  |
| Fornieles et al. (2014)[32] | RCT  N=40 (16 control group)  Sedentary adults with Down syndrome  (23.7±3.1y)  **Group allocation:**  -Resistance exercise  -Control group | **Exercise:**  Exercises of major muscle groups: arm curl, leg extension, seated row, leg curl, triceps extension, and leg press  **Control Group:**  No exercise | **Frequency:**  3d/week  **Intensity:**  40% to 65% 8RM  **Time:**  10 Reps/station first 4 weeks  8 Reps/station last 4 wks.  **Type of Exercise:**  Resistance training  **Length:**  12 weeks | A short-term resistance training protocol improved mucosal immunity response and salivary hormone profile in sedentary adults with Down syndrome. | Low |
|  |  |  |  |  |  |
| Cao Dinh et al. (2019)[33] | RCT  N=100 (36 control group; 31 intensive and 33 strength endurance)  Community dwelling Female older adults (≥65y)  **Group allocation:**  *(mean age* ± *SD)*  -Intensive strength: 69.18±5.12y  - Strength endurance: 69.02±6.05y  - Control group: 70.31±5.15y | **Exercise:**  Seated chest press, seated leg press, seated hip abduction, seated hip adduction, seated low row, and seated vertical traction  **Control Group:**  Stretching exercise | **Frequency:**  2-3d/week  **Intensity:**  Intensive: 80% 1RM  S.Endurance: 40% 1RM  **Time:**  3*10 reps  2*30reps  **Type of Exercise:**  Resistance training  **Length:**  6 weeks | Strength endurance training decreased basal percentage and absolute counts of senescence-prone T cells, which was positively related to the number of training sessions.  Training protocols with many repetitions—at a sufficiently high external resistance—might assist the reduction of senescence-prone T cells in older women. | Moderate |
|  |  |  |  |  |  |
| Ghayomzadeh et al. (2017)[34] | RCT  N= 21 (7 control group)  People with HIV/AIDS  (39±9)  **Group allocation:**  *(mean age* ± *SD)*  -Resistance exercise:38±6y  -Control group: 39±11y | **Exercise:**  - 8 exercises  - Phase 1: elastic band  - Phase 2: bodyweight  **Control Group:**  Usual Care | **Frequency:**  3d/week  **Intensity:**  12-15reps resistance load  **Time:**  NP  **Type of Exercise:**  Resistance training  **Length:**  8 weeks | Resistance exercise program is effective for improving TCD4+ status and body composition in patients with HIV. | Moderate |
|  |  |  |  |  |  |
| Hagstrom et al. (2016)[35] | RCT  N= 39 (19 control group)  Sedentary female breast cancer survivors  (51.9±8.8)  **Group allocation:**  *(mean age* ± *SD)*  -Resistance exercise: 51.2±8.5y  -Control group: 52.7±9.4y | **Exercise:**  Machine based exercises: leg extension, leg curl, lat pulldown, machine bench press, seated roll, back extension, prone hold or sit up  - Free weight barbell squat, deadlift, free-weight barbell bench press, leg press, barbell bent-over row, and assisted chin up)  **Control Group:**  Usual Care | **Frequency:**  3d/week  **Intensity:**  3* 8-10Reps 8RM  (80% 1RM)  **Time:**  60 min  **Type of Exercise:**  Resistance training  **Length:**  16 weeks | Resistance exercise has a beneficial effect on the NK and NKT cell expression of TNF-a indicating that it may be beneficial in improving the inflammatory profile in breast cancer survivors. | Low |
|  |  |  |  |  |  |
| Fahlman et al. (2000)[36] | RCT  N= 29 (14 control group)  Active (Non exercising) Elderly women  **Group allocation:**  *(mean age* ± *SD)*  -Aerobic Exercise: 76±5y  -Control group: 77±6y | **Exercise:**  Walking  **Control Group:**  No exercise | **Frequency:**  3d/week  **Intensity:**  70% HRR  **Time:**  20-50 min  **Type of Exercise:**  Aerobic training  **Length:**  10 weeks | Aerobic training decreased 1-mile walk time and the post-walk heart rate, without resulting in either an acute or chronic suppression of immune function.  Aerobic training may lead to an attenuation of the decrease in cellular immune measures which occurs during the winter. | High |
|  |  |  |  |  |  |
| Bermon et al. (1999)[37] | RCT  N= 32 (16 control group; 16 males)  Elderly sedentary adults  **Group allocation:**  *(mean age* ± *SE)*  - Strength exercise: 70.1±1.0  - Control group: 70.5±0.9 | **Exercise:**  - Leg press, knee extension and seated chest press exercise  **Control Group:**  No exercise | **Frequency:**  3d/week  **Intensity:**  3*8reps 80% 1RM  **Time:** NP  **Type of Exercise:**  Resistance training  **Length:**  8 weeks | 8-week is too short a duration for a strength training programme to modify counts of lymphocyte subsets at rest in elderly sedentary adults. | High |
|  |  |  |  |  |  |
| Baslund et al. (1993)[38] | RCT  N= 18 (9 control group; 16 females)  Patients with rheumatoid arthritis  **Group allocation:**  *(mean age* ± *SE)*  - Exercise group: 49±3y  - Control group: 47±3y | **Exercise:**  - Progressive bicycle training (Monark)  **Control Group:**  No exercise | **Frequency:**  4-5d/week  **Intensity:**  ~80% VO2 max  **Time:**  25 min (3*5min with 5 min each with 10 bpm lower)  **Type of Exercise:**  Aerobic training  **Length:**  8 weeks | 8-week aerobic exercise does not influence the immune system of patients with rheumatoid arthritis. | Moderate |
|  |  |  |  |  |  |
| Hoff et al. (2015)[39] | RCT  N= 24 (9 control group; 24 males)  Medically and psychologically healthy males  **Group allocation:**  *(mean age* ± *SD)*  - Resistive exercise +WBV(n=7):  32.2 ±10.4y  Resistive Exercise(n=8):  31.1 ±5.1y  Control:33.1 ±7.8y | **Exercise:**  - Resistive exercises with whole-body vibration during bed rest  - Resistive exercise only during bed rest  **Control Group:**  No exercise | **Frequency:**  3d/week  **Intensity:**  - 75%-80% of pre-bed rest maximum voluntary contraction - 1.3-1.8 times body **Time:** NP  **Type of Exercise:**  Resistance training  **Length:**  60 days | Prolonged bed rest significantly impacts immune cell populations and cytokine concentrations. Exercise was able to specifically influence different immunological parameters.  The data fit the hypothesis of immuno-protection by exercise and may point toward even superior effects by resistive vibration exercise. | Low |
|  |  |  |  |  |  |
| Cao Ding et al. (2019)[40] | RCT  N=100 (36 control group; 31 intensive and 33 strength endurance)  Cytomegalovirus seropositive and seronegative older women older adults (≥65y)  **Group allocation:**  *(mean age* ± *SD)*  -Intensive strength: 69.18±5.12y  - Strength endurance: 69.02±6.05y  - Control group: 70.31±5.15y | **Exercise:**  Seated chest press, seated leg press, seated hip abduction, seated hip adduction, seated low row, and seated vertical traction  **Control Group:**  Stretching exercise | **Frequency:**  3d/week  **Intensity:**  Intensive: 80% 1RM  S.Endurance: 40% 1RM  **Time:**  3*10 reps  2*30reps  **Type of Exercise:**  Resistance training  **Length:**  6 weeks | Strength endurance training leads to a reduction in circulating senescence-prone T-cells in cytomegalovirus seropositive older women. | High |
|  |  |  |  |  |  |
| Farinatti et al. (2011)[41] | RCT  N=27 (8 control group)  Highly active antiretroviral therapy treated HIV-infected subjects  (45±2y)  **Group allocation:**  *(mean age* ± *SD)*  -Exercise group: 42±5y  - Control group: 46±3y | **Exercise:**  Exercise program consisting of aerobic training, strength and flexibility exercises  **Control Group:**  No exercise | **Frequency:**  3d/week  **Intensity:**  75-85% MHR  **Time:**  90 min  **Type of Exercise:**  Aerobic training, strength and flexibility training (multicomponent)  **Length:**  12 weeks | Overall training can improve the muscle and aerobic fitness of HIV-infected patients with no negative effect on the immunological function. | Low |
|  |  |  |  |  |  |
| Baigis et al. (2002)[42] | RCT  N=123 (55 control group)  People with HIV  (≥18y; 37.0 ±8.1)  **Group allocation:**  -Exercise group  - Control group | **Exercise:**  Home based aerobic exercise (workout on a fitness machine)  **Control Group:**  Social contact (30min visit/wk; Phone calls 2x/wk)" | **Frequency:**  3d/week  **Intensity:**  60-80% of 12 RM  **Time:**  20 min  **Type of Exercise:**  Aerobic training  **Length:**  15 weeks | Aerobic exercise appears to be safe in HIV infected patients. Improvements in physical endurance and quality of life might result if the exercise protocol is longer or progressive. | High |
|  |  |  |  |  |  |
| Woods et al. (1999)[43] | RCT  N=29 (15 control group)  Previously sedentary elderly individuals  (65±0.8y)  **Group allocation:**  -Exercise group  - Control group | **Exercise:**  Moderate intensity aerobic exercises (walking)  **Control Group:**  Muscle group stretching (flexibility) + light resistance exercise against a rubber tubing (toning) | **Frequency:**  3d/week  **Intensity:**  50% to 60-65% VO2max **Time:**  10-15 to 40 min  **Type of Exercise:**  Aerobic training  **Length:**  6 months | 6 months of supervised exercise training can lead to nominal increases in some measures of immune function, while not affecting others, in previously sedentary elderly. |  |
|  |  |  |  |  |  |
| Barrett et al. (2012)[44] | RCT  N=154 (52 control group)    Adults (≥ 50y)  **Group allocation:**  *(mean age* ± *SD)*  - Mindfulness group(n=51): 60.0±6.5y  - Aerobic group (n=51): 59.0±6.6y  - Control group: 58.8±6.8y | **Exercise:**  - Mindfulness meditation  - Moderate intensity sustained exercise (Stationary bicycle, treadmills for group sessions; brisk walking, jogging for home-based activity)  Both with group sessions + home based  **Control Group:**  No exercise | **Frequency:**  1d/week + daily at home practice  **Intensity:**  12-16 point Borg scale  **Time:**  2½hr group session + 45min home practice/activity  **Type of Exercise:**  Aerobic training and mindfulness meditation  **Length:**  8 weeks | Training in meditation or exercise may be effective in reducing acute respiratory Infection illness burden. | Low |
|  |  |  |  |  |  |
| McFarlin et al. (2005)[45] | RCT  N=25 (6 control group)    Older, postmenopausal women (65–85y)  **Group allocation:**  *(mean age* ± *SD)*  -Resistance group: 71.8±6.5y  - Control group: 73.1±6.0y | **Exercise:**  Seated leg press, knee extension, knee flexion, seated chest press, chest flies, lat pull-down, shoulder press, seated rows, hip adduction, and hip abduction  **Control Group:**  No exercise | **Frequency:**  3d/week  **Intensity:**  3sets*8reps in each  70% - 80% RM during  **Time: NP**  **Type of Exercise:**  Resistance training  **Length:**  10 weeks | Increased resting NKCA after chronic resistance training suggests that immunity has been improved. | Moderate |
|  |  |  |  |  |  |
| Shimizu et al. (2007)[46] | RCT  N=48 (20 control group; 20 males)    Healthy sedentary and independently living, elderly subjects  **Group allocation:**  *(mean age* ± *SD)*  -Aerobic + strength group (concurrent): 68.5±0.7y  - Control group: 69.8±1.1y | **Exercise:**  Cycle ergometer + body weight against gravity exercises (squat, trunk-curl, back-extension, leg-curl, and calf-raise)  **Control Group:**  No exercise | **Frequency:**  5d/week  **Intensity:**  80% work rate double-product breakpoint  3sets*10reps  **Time:**  30 min aerobic part  **Type of Exercise:**  Concurrent training  **Length:**  6 weeks | Concurrent exercise training in the elderly is associated with improvement  of expression of CD28 on Th cells and Th1/Th2 balances. Therefore, exercise training could up-regulate Th cell-mediated immune functions and be helpful for a decrease in the risk of infections and autoimmune diseases in elderly people. | Moderate |
|  |  |  |  |  |  |
| McDowell et al. (1992)[47] | RCT  N=30 (6 control group; 12 Low intensity Training; 12 High intensity training)    Healthy adult males  (22.1±3.0y)  **Group allocation:**  *(mean age* ± *SD)*  - Low intensity: 23.3±3.9y  - High intensity: 22.6±2.9y  - Control group: 20.3±3.3y | **Exercise:**  Running  **Control Group:**  No exercise | **Frequency:**  3d/week  **Intensity:**  70% and at 86% MHR for low and high intensities.  **Time:**  20 min  **Type of Exercise:**  Aerobic training  **Length:**  10 weeks | s-IgA response to maximal exercise was unaffected by low to high intensity training and independent of salivary cortisol. | Low |
|  |  |  |  |  |  |
| Nehlsen-Cannarella et al. (1991)[48] | RCT  N=36 (18 each group)    sedentary middle obese women  **Group allocation:**  *(mean age* ± *SE)*  - Exercise group: 36.0±1.6y  - Control group: 32.8±1.4y | **Exercise:**  Brisk walking  **Control Group:**  No exercise | **Frequency:**  5d/week  **Intensity:**  60% HRR  **Time:**  45 min  **Type of Exercise:**  Aerobic training  **Length:**  15 weeks | Moderate aerobic exercise is not associated with an improvement in lymphocytes function but is associated with a 20% increase in serum immunoglobulins and several small changes in circulating numbers of immune system variables, highlighted by decreases in circulating numbers of lymphocytes, particularly the T cell subpopulation. | Moderate |
|  |  |  |  |  |  |
| Nieman et al. (1990)[49] | RCT  N=36 (18 each group;)    Mildly obese sedentary women (25-45y)  **Group allocation:**  *(mean age* ± *SE)*  - Exercise group: 36.0±1.6y  - Control group: 32.8±1.4y | **Exercise:**  Brisk walking  **Control Group:**  No exercise | **Frequency:**  5d/week  **Intensity:**  60% HRR  **Time:**  45 min  **Type of Exercise:**  Aerobic training  **Length:**  15 weeks | Moderate aerobic exercise is associated with elevated NK cell activity after six but not 15 weeks, and reduced upper respiratory tract infection symptomatology in comparison to a randomised, sedentary control group. | Low |
|  |  |  |  |  |  |

Table S4. Descriptive characteristics of the 6 vaccination studies included.

| **Study (Year)** | **Design and Sample** | **Exercise/PA and control conditions** | **Intervention Parameters (F.I.T.T.)** | **Main finding/Conclusions** | **Risk of Bias** |
| --- | --- | --- | --- | --- | --- |
| Irwin et al. (2007)[50] | RCT  N=112 (53 control group; 41 males)    Healthy older adult  (59-86y)  **Group allocation:**  *(mean age* ± *SE)*  - Tai Chi Chi group: 69.6±6.2y  - Control group: 70.2 ±7.5y | **Exercise:**  20 exercises according to therapist manual and supervised by master level instructors.  **Control Group:**  Health Education (120 min/week)  16 didactic presentations on health-related themes. | **Frequency:**  3d/week  **Intensity:** NP  **Time:**  40 min  **Type of Exercise:**  Mobility (Tai Chi Chi) training  **Length:**  16 weeks + follow up to 25 weeks (end of the post-vaccination period) | Tai Chi augments resting levels of varicella zoster virus specific cell mediated-immunity and boosts varicella zoster virus - cell mediated-immunity of the **varicella vaccine**. | Moderate |
|  |  |  |  |  |  |
| Kohut et al. (2004)[51] | RCT  N=27 (13 control group)    Older adult  (>64y)  **Group allocation:**  *(mean age* ± *SD)*  - Exercise group:73.07±5.6y  - Control group: 70.25±5.6y | **Exercise:**  Treadmills, stair-stepping, rowing machine, cycle ergometer  **Control Group:**  No Exercise | **Frequency:**  3d/week  **Intensity:**  from 40-60%HRR  To 65-75%HRR  **Time:**  20-30 min  **Type of Exercise:**  Aerobic training  **Length:**  10 months | Aerobic exercise may enhance the mean fold increase in antibody titre in response to **influenza** immunisation if the influenza antigen was contained in the previous year’s vaccine. | High |
|  |  |  |  |  |  |
| Woods et al. (2009)[52] | RCT  N=160 (82 control group)    Sedentary healthy older adults (69.9±0.4y)  **Group allocation:**  *(mean age* ± *SD)*  - Exercise group:69.6±4.9y  - Control group: 70.1±5.7y | **Exercise:**  Walking, cycling, elliptical, stair climbing  **Control Group:**  muscle stretching + balance training | **Frequency:**  3d/week  **Intensity:**  60-70% VO2max  **Time:**  45-60 min  **Type of Exercise:**  Aerobic training  **Length:**  10 months | Aerobic exercise experienced improvements in **influenza** seroprotection throughout the entire influenza season, but not controls. Although there were no differences in reported respiratory tract infections, the exercise group exhibited reduced overall illness severity and sleep disturbance. | Low |
|  |  |  |  |  |  |
| Long et al. (2013)[53] | RCT  N= 89 (45 control group)  Sedentary women  (45.6 ± 1.1 y)  **Group allocation:**  *(mean age +/- SD)*  -Exercise group: 47.84±6.56y  -Control Group: 46.87±7.38y | **Exercise:**  Physical activity consultation, pedometer, telephone/e-mail prompts  **Control Group:**  Advisory leaflet on  physical activity | **Frequency:** NP  **Intensity:** NP  **Time:** NP  **Type of Exercise:**  Aerobic training  **Length:**  16 weeks (+6-month follow-up) | A life-style physical activity intervention increased subjective and objective physical activity levels and quality of life but did not affect antibody response to **pneumococcal vaccination**. | Low |
|  |  |  |  |  |  |
| Kohut et al. (2005)[54] | RCT  N= 28 (14 each group; 14 females in total)  Older adults after immunisation (aged 73± 5.6)  **Group allocation:**  *(mean age* ± *SD)*  - Exercise group:73.07±5.59y  - Control group: 70.25±5.57y | **Exercise:**  Treadmills, stair-stepping, rowing machine, cycle ergometer  **Control Group:**  No Exercise | **Frequency:**  3d/week  **Intensity:**  from 40-60%HRR  To 65-75%HRR  **Time:**  20-30 min  **Type of Exercise:**  Aerobic training  **Length:**  10 months | Aerobic exercise shows promise as a positive immunomodulator in older adults after immunisation. | Moderate |
|  |  |  |  |  |  |
| Hayney et al. (2014)[55] | RCT  N= 154; 27 males.  (N=47 exercise, N=51 meditation, N=51 control)  Healthy Overweight adults  60±7y (50-76y)  **Group allocation:**  *(mean age* ± *SD)(range)*  - Exercise group: 59±6.6y (range 50-73y)  -Meditation group: 60±6.5y (range 50-72y)  - Control group: 58.8±6.8y (range 50-76y) | **Exercise:**  Senior exercise programme  **Control Group:**  No Exercise | **Frequency:**  2d/week  **Intensity:**  12-16 Borg scale (MVPA)  **Time:**  90 min  **Type of Exercise:**  NP  **Length:**  8 weeks | The meditation and exercise training failed to enhance immune responses to **influenza vaccine**. However, optimism, perceived stress, and anxiety were correlated in the expected directions with antibody responses to influenza vaccine. | High |

**Risk of bias assessment details**

Table S5: Detail of the risk of bias assessment for observational studies.

| Study | Risk of bias domains | | | | | | | Overall risk of bias |
| --- | --- | --- | --- | --- | --- | --- | --- | --- |
|  | Confounding | Selection | Classification of exposure | Departure | Missing data | Measurement | Reporting bias |  |
| Hamer et al 2019 | Moderate | Low | Moderate | Moderate | Moderate | Low | Low | Moderate |
| Baik et al 2000 | Moderate | Low | Moderate | Moderate | Moderate | Low | Low | Moderate |
| Inoue et al 2007 | Moderate | Low | Moderate | Moderate | Moderate | Low | Moderate | Moderate |
| Paulsen et al 2017 | Moderate | Low | Moderate | Moderate | Moderate | Low | Low | Moderate |
| Wang et al 2017 | Low | Low | Low | Moderate | Moderate | Low | Low | Moderate |
| Williams et al 2014 | High | Low | Moderate | Moderate | High | Low | Low | High |

Table S6: Detail of risk of bias assessment for experimental physical activity intervention studies

| Study | Risk of bias domains | | | | | | Overall bias |
| --- | --- | --- | --- | --- | --- | --- | --- |
|  | Randomisation | Deviation | Missing data | Measurement | Selection | |  |
| Abd El-Kader et al (2018) | Some concerns | Some concerns | High | High | Some concerns | | High |
| Campbell et al (2008) | Low | Some concerns | Low | Some concerns | Some concerns | | Moderate |
| Kim et al (2006) | Low | Low | Low | Low | Low | | Low |
| Hwang et al (2016) | High | High | High | Some concerns | Low | | High |
| Wang et al (2011) | Low | Some concerns | Low | High | Some concerns | | High |
| Weng et al (2013) | Some concerns | Some concerns | Low | Some concerns | Some concerns | | Moderate |
| Ezema et al (2014) | Some concerns | Some concerns | Low | High | High | | High |
| Schmidt et al (2018) | Low | Some concerns | Low | Low | High | | High |
| Anandh et al (2013) | Low | Low | Low | Low | Some concerns | | Low |
| Fornieles et al (2014) | Some concerns | Low | Low | Low | Low | | Low |
| Cao Dinh et al (2019) | Low | Low | Some concerns | Low | Some concerns | | Moderate |
| Ghayomzadeh et al (2017) | Some concerns | Some concerns | Some concerns | Low | Some concerns | | Moderate |
| Hagstrom et al (2016) | Low | Low | Low | Low | Low | | Low |
| Fahlman et al (2000) | Some concerns | Some concerns | Some concerns | Some concerns | Some concerns | | High |
| Bermon et al (1999) | Some concerns | Some concerns | Some concerns | Some concerns | Some concerns | | High |
| Baslund et al (1993) | Some concerns | Some concerns | Low | Low | Some concerns | | Moderate |
| Hoff et al (2015) | Some concerns | Low | Low | Low | Low | | Low |
| Cao Dinh et al (2019) | Some concerns | Some concerns | Low | Some concerns | Some concerns | | High |
| Farinatti et al (2010) | Some concerns | Low | Low | Low | Low | | Low |
| Baigis et al (2002) | Some concerns | High | High | Low | Some concerns | | High |
| Woods et al (1999) | Some concerns | Some concerns | Low | Low | Some concerns | | Moderate |
| Hoffman-Goetz el al (1990) | Some concerns | Some concerns | Low | Some concerns | High | | High |
| Barrett et al (2012) | Low | Some concerns | Low | Low | Low | | Low |
| McFarlin et al (2005) | Some concerns | Low | Some concerns | Low | Some concerns | | Moderate |
| Shimizu et al (2008) | Some concerns | Low | Low | Low | Some concerns | | Moderate |
| McDowell et al (1992) | Low | Low | Some concerns | Low | Low | | Low |
| Shimizu et al (2011) | High | Some concerns | Some concerns | Low | Some concerns | | High |
| Mitchell et al (1996) | Low | Some concerns | Low | Low | Some concerns | | Moderate |
| Shimizu et al (2007)b | Some concerns | Some concerns | Low | Low | Some concerns | | Moderate |
| Wang et al (2011)b | Some concerns | Low | Some concerns | Some concerns | Low | | Moderate |
| Ciloglu et al (2005) | Some concerns | Some concerns | Low | Low | Low | | Moderate |
| Nehlsen-Cannarella et al (1991) | Some concerns | Some concerns | Low | Low | Low | | Moderate |
| Unal et al (2005) | Some concerns | Some concerns | Low | Low | Low | | Moderate |
| Flynn et al (1999) | Some concerns | Some concerns | Low | Low | Low | | Moderate |
| Nieman et al (1990) | Some concerns | Low | Low | Low | Low | | Low |
| Nieman et al (1998) | Some concerns | Low | Low | Low | Low | | Low |
| Wang et al (2011)b | Some concerns | Some concerns | Low | Low | Some concerns | | Moderate |
| Hutnick et al (2005) | Some concerns | Low | Low | Low | Some concerns | | Moderate |
| Martins et al (2009) | Some concerns | Some concerns | Low | Low | | Some concerns | Moderate |
| Makras et al (2005) | High | High | Low | Low | | Low | High |
| Klentrou et al (2002) | Some concerns | Low | Low | Low | | Some concerns | Moderate |
| Fahlman et al (2003) | Low | Some concerns | Some concerns | Low | | Low | Moderate |

Table S7: Detail of risk of bias assessment for vaccination studies

| Study | Risk of bias domains | | | | | Overall bias |
| --- | --- | --- | --- | --- | --- | --- |
|  | Randomisation | Deviation | Missing data | Measurement | Selection |  |
| Kohut et al (2005) | Some concerns | Some concerns | Low | Low | Low | Moderate |
| Hayney et al (2014) | Low | Some concerns | Low | Some concerns | High | High |
| Kohut et al (2004) | Some concerns | Some concerns | Some concerns | Some concerns | Some concerns | High |
| Woods et al (2009) | Low | Low | Low | Low | Low | Low |
| Irwin et al (2007) | Low | Low | Some concerns | Low | Some concerns | Moderate |
| Long et al (2013) | Some concerns | Low | Low | Low | Low | Low |

Additional Meta-analysis results


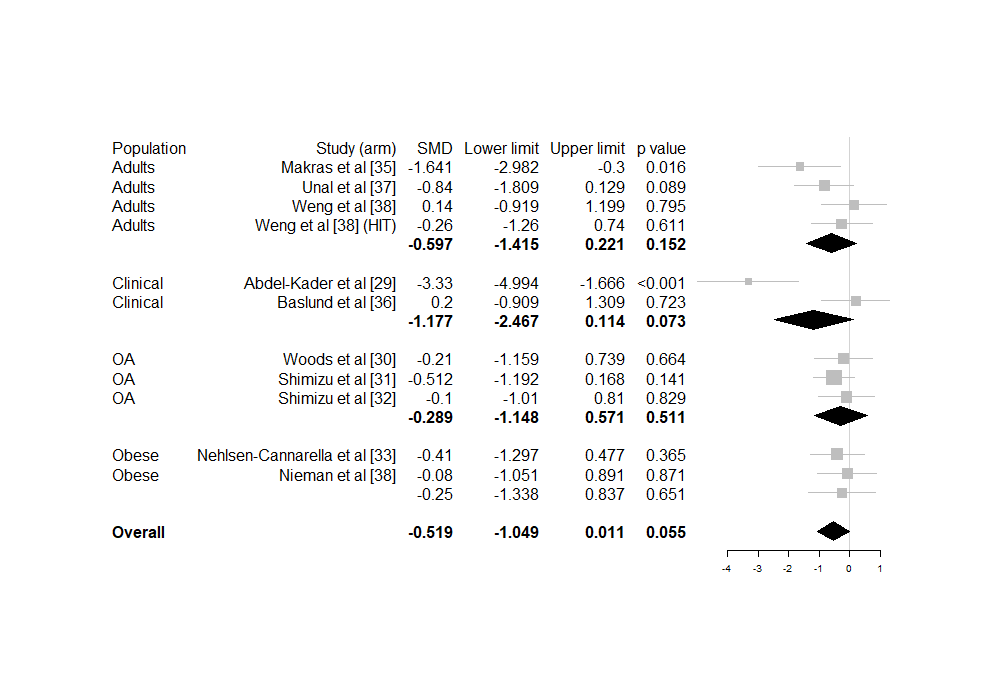


Figure S1: : Forest plot for total white blood cell count (Leukocytes) in healthy adults, clinical groups, obese adults and older adults (OA). Mean difference (MD) is in cell/pL. Size of the square represent the weight of each study in the meta-analysis. References refer to main manuscript referencing for comparison with other forest plots.


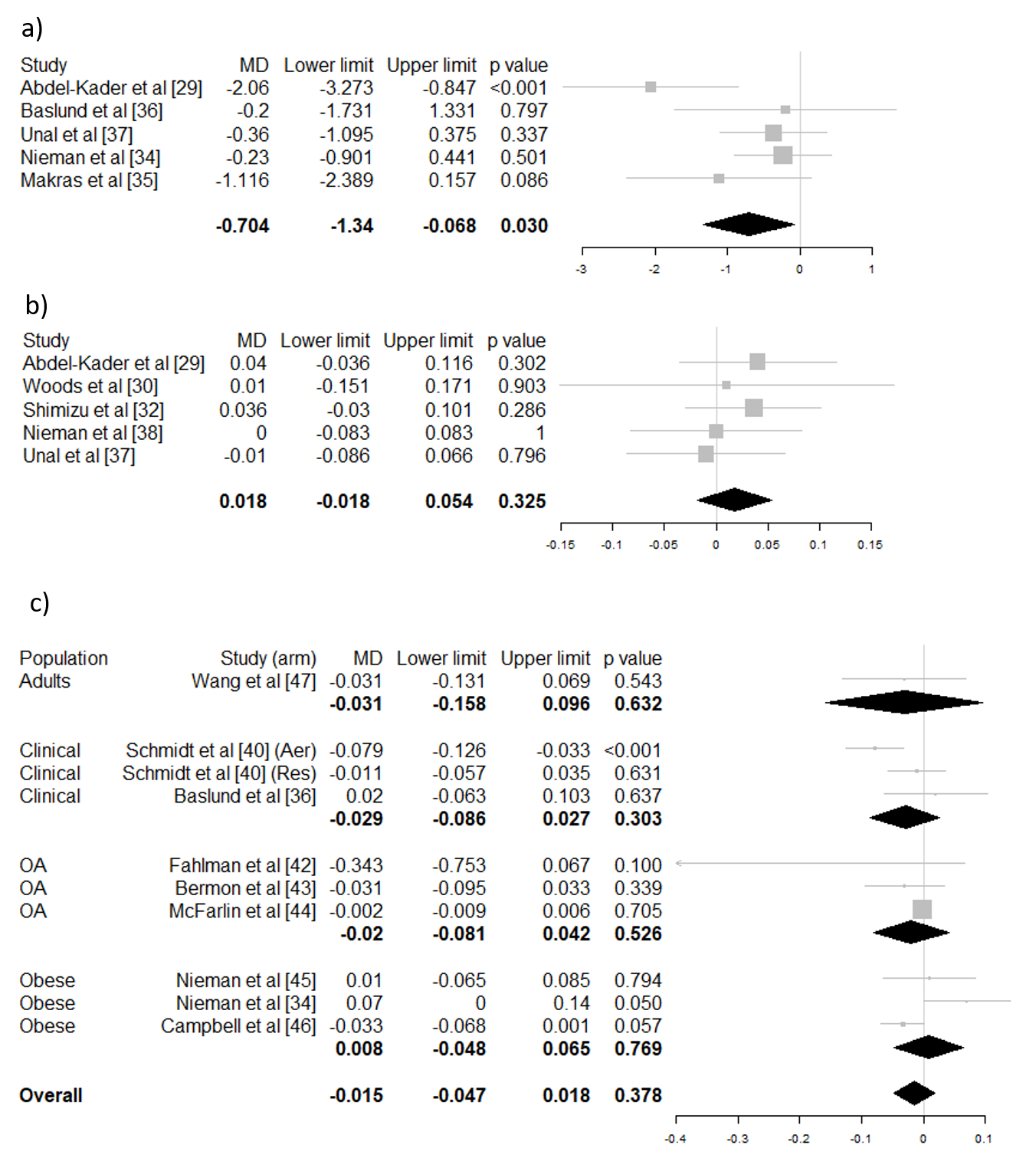


Figure S2: Forest plot for innate immune system cell counts a) Neutrophils b) Monocytes c) Natural killer in healthy adults, clinical groups, obese adults and older adults (OA). Mean difference (MD) is in cell/pL. Size of the square represent the weight of each study in the meta-analysis. References refer to main manuscript referencing for comparison with other forest plots.


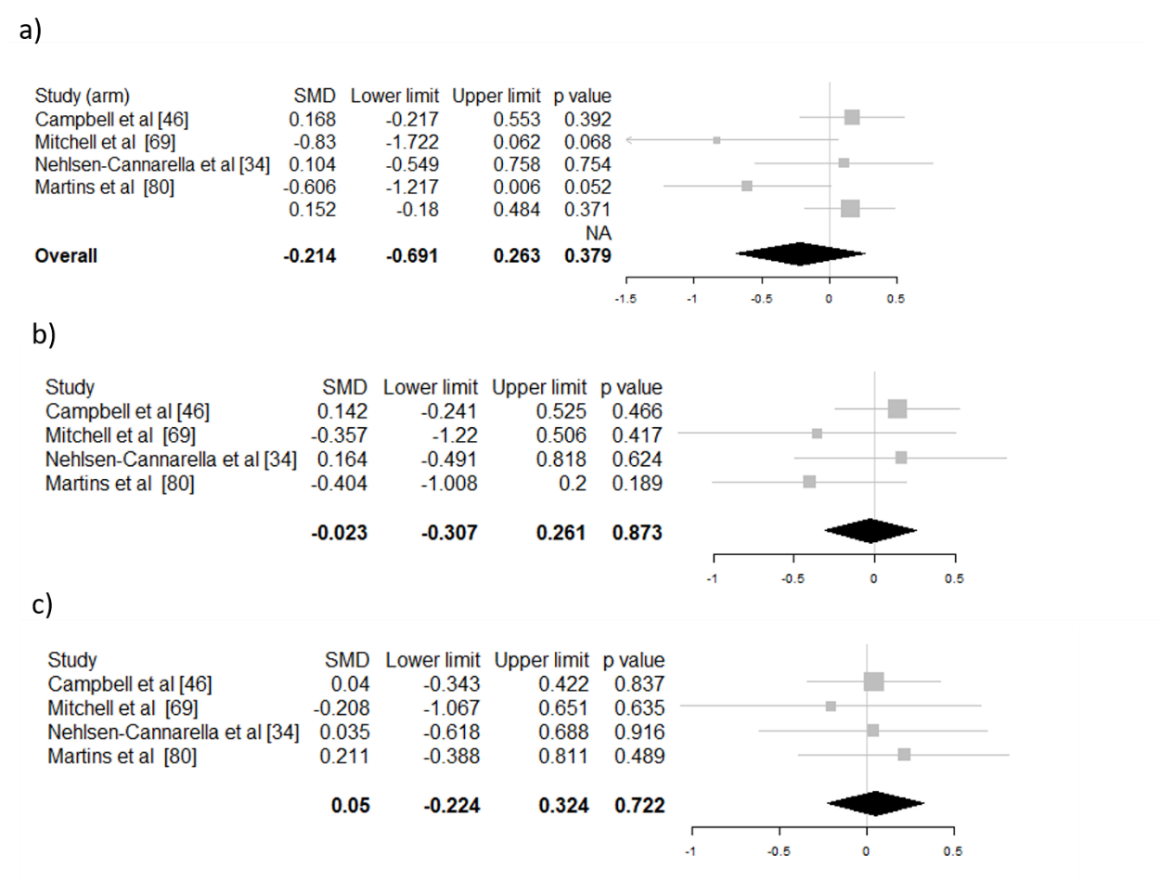

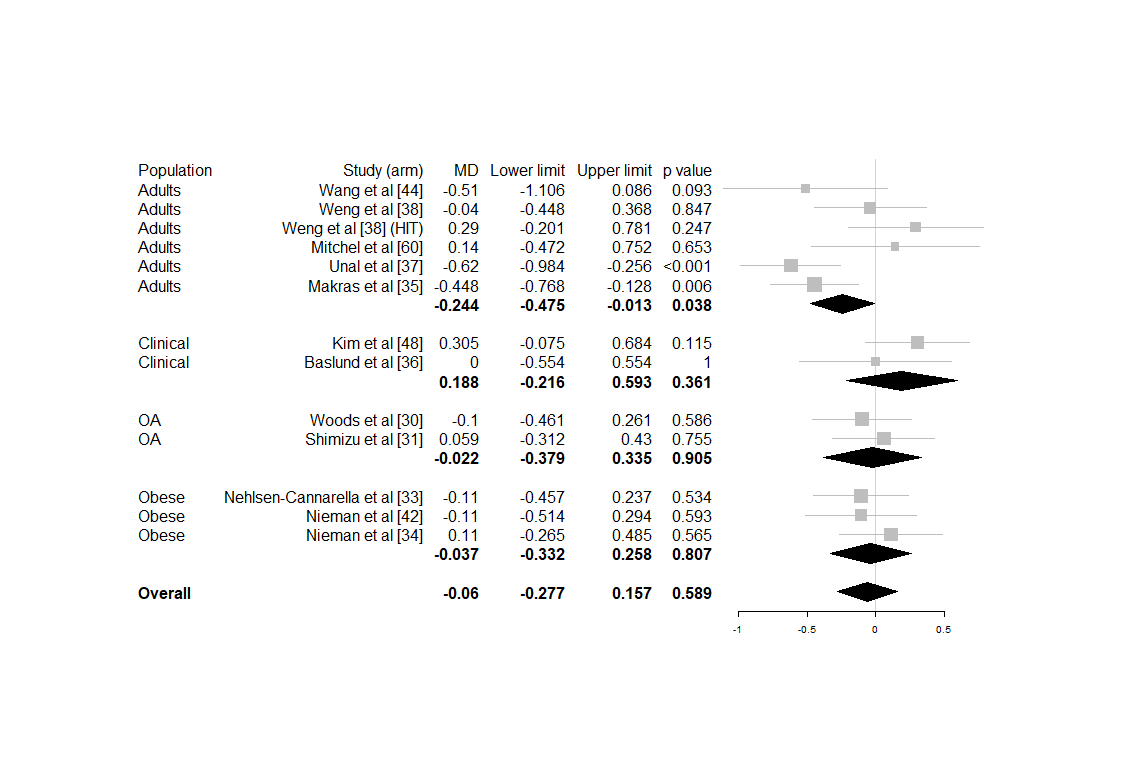


Figure S4: Forest plot for immunoglobulin concentration of a) serum IgA (SIgA) b) IgG and c) IgM for healthy adults, clinical groups, obese adults and older adults (OA). Size of the square represent the weight of each study in the meta-analysis. References refer to main manuscript referencing for comparison with other forest plots.

Figure S3: Forest plot for lymphocytes counts in healthy adults, clinical groups, obese adults and older adults (OA). Mean difference (MD) is in cell/pL. Size of the square represent the weight of each study in the meta-analysis. References refer to main manuscript referencing for comparison with other forest plots.

**Publication bias**

Figure S5: Funnel plot for experimental studies reporting Leukocytes counts

Figure S6: Funnel plot for observational studies

Figure S7: Funnel plot for experimental studies reporting Neutrophil counts.

Figure S8: Funnel plot for experimental studies reporting monocytes counts.

Figure S9: Funnel plot for studies reporting lymphocytes counts.

Figure S10: Funnel plot for experimental studies reporting Natural Killer counts.

Figure S11: Funnel plot for experimental studies reporting CD3+ counts.

Figure S12: Funnel plot for studies reporting CD4 counts.

Figure S13: Funnel plot for studies reporting CD8 counts.

Figure S14: Funnel plot for experimental studies reporting IgA.

Figure S15: Funnel plot for vaccination studies

References

1 Baik I, Curhan GC, Rimm EB, *et al.* A prospective study of age and lifestyle factors in relation to community-acquired pneumonia in US men and women. *Arch Intern Med* 2000;**160**:3082–8. doi:10.1001/archinte.160.20.3082

2 Hamer M, O’Donovan G, Stamatakis E. Lifestyle risk factors, obesity and infectious disease mortality in the general population: Linkage study of 97,844 adults from England and Scotland. *Prev Med (Baltim)* 2019;**123**:65–70. doi:10.1016/j.ypmed.2019.03.002

3 Paulsen J, Askim Å, Mohus RM, *et al.* Associations of obesity and lifestyle with the risk and mortality of bloodstream infection in a general population: a 15-year follow-up of 64 027 individuals in the HUNT Study. *Int J Epidemiol* 2017;**46**:1573–81. doi:10.1093/ije/dyx091

4 Wang HE, Baddley J, Griffin RL, *et al.* Physical inactivity and long-term rates of community-acquired sepsis. *Prev Med (Baltim)* 2014;**65**:58–64. doi:http://dx.doi.org/10.1016/j.ypmed.2014.04.017

5 Inoue Y, Koizumi A, Wada Y, *et al.* Risk and protective factors related to mortality from pneumonia among middleaged and elderly community cesidents: The JACC study. *J Epidemiol* 2007;**17**:194–202. doi:10.2188/jea.17.194

6 Williams PT. Dose-response relationship between exercise and respiratory disease mortality. *Med Sci Sports Exerc* 2014;**46**:711–7. doi:10.1249/MSS.0000000000000142

7 Neuman MI, Willett WC, Curhan GC. Physical activity and the risk of community-acquired pneumonia in US women. *Am J Med* 2010;**123**:281.e7–281.e11. doi:10.1016/j.amjmed.2009.07.028

8 Wang J-S, Chen W-L, Weng T-P. Hypoxic exercise training reduces senescent T-lymphocyte subsets in blood. *Brain Behav Immun* 2011;**25**:270–8. doi:10.1016/j.bbi.2010.09.018

9 Hoffman-Goetz L, Simpson JR, Cipp N, *et al.* Lymphocyte subset responses to repeated submaximal exercise in men. *J Appl Physiol* 1990;**68**:1069–74.

10 Ciloğlu F. The effect of exercise on salivary IgA levels and the incidence of upper respiratory tract infections in postmenopausal women. *Kulak burun bogaz Ihtis Derg* 2005;**15**:112–6.

11 Unal M, Erdem S, Deniz G. The effects of chronic aerobic and anaerobic exercises on lymphocyte subgroups. *Acta Physiol Hung* 2005;**92**:163–71.

12 Flynn MG, Fahlman M, Braun WA, *et al.* Effects of resistance training on selected indexes of immune function in elderly women. *J Appl Physiol* 1999;**86**:1905–13.

13 Nieman DC, Nehlsen-Cannarella SL, Henson DA, *et al.* Immune response to exercise training and/or energy restriction in obese women. *Med Sci Sports Exerc* 1998;**30**:679–86.

14 Wang M-Y, An L-G. EFFECTS OF 12 WEEKS’ TAI CHI CHUAN PRACTICE ON THE IMMUNE FUNCTION OF FEMALE COLLEGE STUDENTS WHO LACK PHYSICAL EXERCISE. *Biol Sport* 2011;**28**:45–9. doi:10.5604/935875

15 Hutnick N, Williams N, Kraemer W, *et al.* Exercise and lymphocyte activation following chemotherapy for breast cancer. *Med Sci Sport Exerc* 2005;**37**:1827–35.

16 Martins R, Cunha M, Neves A, *et al.* Effects of aerobic conditioning on salivary IgA and plasma IgA, IgG and IgM in older men and women. *Int J Sports Med* 2009;**30**:906–12. doi:10.1055/s-0029-1237389

17 Makras P, GN K, Bourikas G, *et al.* Effect of 4 weeks of basic military training on peripheral blood leucocytes and urinary excretion of catecholamines and cortisol. *J Sports Sci* 2005;**23**:825–34.

18 Klentrou P, Cieslak T, Macneil M, *et al.* Effect of moderate exercise on salivary immunoglobulin A and infection risk in humans. *Eur J Appl Physiol* 2002;**87**:153–8. doi:http://dx.doi.org/10.1007/s00421-002-0609-1

19 Shimizu K, Kimura F, Akimoto T, *et al.* Effect of moderate exercise training on T-helper cell subpopulations in elderly people. *Exerc Immunol Rev* 2011;**14**:24–37.

20 Mitchell J, Paquet J, Pizza F, *et al.* The effect of moderate aerobic training on lymphocyte proliferation. *Int J Sport Med* 1996;**17**:384–9. doi:10.1055/s-2007-972865

21 Shimizu K, Kimura F, Akimoto T, *et al.* Effects of exercise, age and gender on salivary secretory immunoglobulin A in elderly individuals. *Exerc Immunol Rev* 2007;**13**:55–66.

22 Abd El-Kader SM, Al-Shreef FM. Impact of aerobic exercises on selected inflammatory markers and immune system response among patients with sickle cell anemia in asymptomatic steady state. *Afr Health Sci* 2018;**18**:111–9. doi:10.4314/ahs.v18i1.15

23 Campbell PT, Wener MH, Sorensen B, *et al.* Effect of exercise on in vitro immune function: a 12-month randomized, controlled trial among postmenopausal women. *J Appl Physiol* 2008;**104**:1648–55. doi:10.1152/japplphysiol.01349.2007

24 Kim S-D, Kim H-S. A series of bed exercises to improve lymphocyte count in allogeneic bone marrow transplantation patients. *Eur J Cancer Care (Engl)* 2006;**15**:453–7. doi:10.1111/j.1365-2354.2006.00668.x

25 Hwang Y, Park J, Lim K. Effects of Pilates Exercise on Salivary Secretory Immunoglobulin A Levels in Older Women. *J Aging Phys Act* 2016;**24**:399–406. doi:10.1123/japa.2015-0005

26 Wang J-S, Weng T-P. Hypoxic exercise training promotes antitumour cytotoxicity of natural killer cells in young men. *Clin Sci* 2011;**121**:343–53. doi:10.1042/CS20110032

27 Weng T-P, Huang S-C, Chuang Y-F, *et al.* Effects of interval and continuous exercise training on CD4 lymphocyte apoptotic and autophagic responses to hypoxic stress in sedentary men. *PLoS One* 2013;**8**:e80248. doi:10.1371/journal.pone.0080248

28 Ezema CI, Onwunali AA, Lamina S, *et al.* Effect of aerobic exercise training on cardiovascular parameters and CD4 cell count of people living with human immunodeficiency virus/acquired immune deficiency syndrome: A randomized controlled trial. *Niger J Clin Pract* 2014;**17**:543–8. doi:10.4103/1119-3077.141414

29 Schmidt T, Jonat W, Wesch D, *et al.* Influence of physical activity on the immune system in breast cancer patients during chemotherapy. *J Cancer Res Clin Oncol* 2018;**144**:579–86. doi:10.1007/s00432-017-2573-5

30 Fahlman MM, Morgan AL, McNevin N, *et al.* Salivary s-IgA response to training in functionally fimited elders. *J Aging Phys Act* 2003;**11**:502‐515.

31 Anandh V, D’Sa IP, Alagesan J. Effect of progressive resistance training on cardio vascular fitness, quality of life and CD4 count in people with HIV/AIDS. *Glob J Res Anal* 2014;**3**:555‐559.

32 Fornieles G, Rosety MA, Elosegui S, *et al.* Salivary testosterone and immunoglobulin A were increased by resistance training in adults with Down syndrome. *Brazilian J Med Biol Res* 2014;**47**:345‐348. doi:10.1590/1414-431x20143468

33 Cao Dinh H, Bautmans I, Beyer I, *et al.* Six weeks of strength endurance training decreases circulating senescence-prone T-lymphocytes in cytomegalovirus seropositive but not seronegative older women. *Immun Ageing* 2019;**16**:17. doi:10.1186/s12979-019-0157-8

34 Ghayomzadeh M, SeyedAlinaghi S, Shamsi MM, *et al.* Effect of 8 Weeks of Hospital-Based Resistance Training Program on TCD4+ Cell Count and Anthropometric Characteristic of Patients With HIV in Tehran, Iran: a Randomized Controlled Trial. *J Strength Cond Res* 2017;**33**:1146‐1155. doi:10.1519/JSC.0000000000002394

35 Hagstrom AD, Marshall PW, Lonsdale C, *et al.* The effect of resistance training on markers of immune function and inflammation in previously sedentary women recovering from breast cancer: a randomized controlled trial. *Breast Cancer Res Treat* 2016;**155**:471‐482. doi:10.1007/s10549-016-3688-0

36 Fahlman M, Boardley D, Flynn MG, *et al.* Effects of endurance training on selected parameters of immune function in elderly women. *Gerontology* 2000;**46**:97‐104. doi:10.1159/000022142

37 Bermon S, Philip P, Ferrari P, *et al.* Effects of a short-term strength training programme on lymphocyte subsets at rest in elderly humans. *Eur J Appl Physiol Occup Physiol* 1999;**79**:336‐340. doi:10.1007/s004210050517

38 Baslund B, Lyngberg K, Andersen V, *et al.* Effect of 8 wk of bicycle training on the immune system of patients with rheumatoid arthritis. *J Appl Physiol* 1993;**75**:1691–5. doi:10.1152/jappl.1993.75.4.1691

39 Hoff P, Belavý DL, Huscher D, *et al.* Effects of 60-day bed rest with and without exercise on cellular and humoral immunological parameters. *Cell Mol Immunol* 2015;**12**:483‐492. doi:10.1038/cmi.2014.106

40 Cao Dinh H, Njemini R, Onyema OO, *et al.* Strength endurance training but not intensive strength training reduces senescence-prone T cells in peripheral blood in community-dwelling elderly women. *Journals Gerontol - Ser A Biol Sci Med Sci* 2019;**74**:1870‐1878. doi:10.1093/gerona/gly229

41 Farinatti PT, Borges JP, Gomes RD, *et al.* Effects of a supervised exercise program on the physical fitness and immunological function of HIV-infected patients. *J Sports Med Phys Fitness* 2011;**50**:511‐518.

42 Baigis J, Korniewicz DM, Chase G, *et al.* Effectiveness of a home-based exercise intervention for HIV-infected adults: a randomized trial. *J Assoc Nurses AIDS care* 2002;**13**:33‐45. doi:10.1016/S1055-3290(06)60199-4

43 Woods JA, Ceddia MA, Wolters BW, *et al.* Effects of 6 months of moderate aerobic exercise training on immune function in the elderly. *Mech Ageing Dev* 1999;**109**:1–19. doi:10.1016/S0047-6374(99)00014-7

44 Barrett B, Hayney MS, Muller D, *et al.* Meditation or exercise for preventing acute respiratory infection: A randomized controlled trial. *Ann Fam Med* 2012;**10**:337–46. doi:10.1370/afm.1376

45 McFarlin BK, Flynn MG, Phillips MD, *et al.* Chronic resistance exercise training improves natural killer cell activity in older women. *Journals Gerontol - Ser A Biol Sci Med Sci* 2005;**60**:1315–8. doi:10.1093/gerona/60.10.1315

46 Shimizu K, Suzuki N, Imai T, *et al.* Monocyte and T-cell responses to exercise training in elderly subjects. *J Strength Cond Res* 2007;**25**:2565–72. doi:10.1519/JSC.0b013e3181fc5e67

47 McDowell SL, Hughes RA, Hughes RJ, *et al.* The effect of exercise training on salivary immunoglobulin A and cortisol responses to maximal exercise. *Int J Sports Med* 1992;**13**:577–80.

48 Nehlsen-Cannarella SL, Nieman DC, Balk-Lamberton AJ, *et al.* The effects of moderate exercise training on immune response. *Med Sci Sports Exerc* 1991;**23**:64–70.

49 Nieman D, Nehlsen-Cannarella S, Markoff P, *et al.* The effects of moderate exercise training on natural killer cells and acute upper respiratory tract infections. *Int J Sports Med* 1990;**11**:467–73. doi:10.1055/s-2007-1024839

50 Irwin M, Olmstead R, Oxman M. Augmenting immune responses to varicella zoster virus in older adults: a randomized, controlled trial of Tai Chi. *J Am Geriatr Soc* 2007;**55**:511–7. doi:10.1111/j.1532-5415.2007.01109.x

51 Kohut ML, Arntson BA, Lee WL, *et al.* Moderate exercise improves antibody response to influenza immunization in older adults. *Vaccine* 2004;**22**:2298–306. doi:10.1016/j.vaccine.2003.11.023

52 Woods JA, Keylock KT, Lowder T, *et al.* Cardiovascular exercise training extends influenza vaccine seroprotection in sedentary older adults: The immune function intervention trial. *J Am Geriatr Soc* 2009;**57**:2183–91. doi:10.1111/j.1532-5415.2009.02563.x

53 Long JE, Ring C, Bosch JA, *et al.* A life-style physical activity intervention and the antibody response to pneumococcal vaccination in women. *Psychosom Med* 2013;**75**:774–82. doi:10.1097/PSY.0b013e3182a0b664

54 Kohut ML, Lee W, Martin A, *et al.* The exercise-induced enhancement of influenza immunity is mediated in part by improvements in psychosocial factors in older adults. *Brain Behav Immun* 2005;**19**:357–66. doi:10.1016/j.bbi.2004.12.002

55 Hayney MS, Coe CL, Muller D, *et al.* Age and psychological influences on immune responses to trivalent inactivated influenza vaccine in the meditation or exercise for preventing acute respiratory infection (MEPARI) trial. *Hum Vaccin Immunother* 2014;**10**:83–91. doi:10.4161/hv.26661

# Declarations

**Funding:** No specific funding for this study. We would like to thank Veritas Health Innovation for granting us free access to the COVIDENCE software for this study under the COVID-19 team initiative. DJP was supported by a grant from the Spanish Ministry of Science and Innovation - MINECO (RYC-2014-16938).

**Conflicts of interest/Competing interests:** Sebastien Chastin, Ukachukwu Abaraogu, Jan Bourgois, Philippa Dall, Jennifer Darnborough, Elain Duncan, Jasmien Dumortier, David Jiménez Pavón, Joanna McParland, Nicola Roberts and Mark Hamer declare that they have no conflicts of interest relevant to the content of this review.

**Ethics approval:** Not applicable.

**Consent:** Not applicable.

### Availability of data and material: The datasets generated during and/or analysed during the current review are available from the corresponding author on reasonable request.

**Authors’ Contributions:** Substantial contributions to the conception or design of the work (SC, UA, and MH); acquisition of the data (all authors); data extraction (all authors); statistical analyses (SC); interpretation of the data (all authors); drafting the work (SC, MH, JB, JD); revising it critically for important intellectual content (all authors); final approval of the version to be published (all authors); agreement to be accountable for all aspects of the work (all authors). The corresponding author attests that all listed authors meet authorship criteria and that no others meeting the criteria have been omitted.
